# Ginsenoside Rk2 alleviates hepatic ischemia/reperfusion injury by enhancing AKT membrane translocation and activation

**DOI:** 10.1002/mco2.70047

**Published:** 2025-01-14

**Authors:** Hong Shen, Jiajun Fu, Jiayue Liu, Toujun Zou, Kun Wang, Xiao‐Jing Zhang, Jian‐Bo Wan

**Affiliations:** ^1^ State Key Laboratory of Quality Research in Chinese Medicine Institute of Chinese Medical Sciences University of Macau Macao SAR China; ^2^ State Key Laboratory of New Targets Discovery and Drug Development for Major Diseases Gannan Innovation and Translational Medicine Research Institute Gannan Medical University Ganzhou China; ^3^ Department of Cardiology Renmin Hospital of Wuhan University Wuhan China; ^4^ Basic Medical School Wuhan University Wuhan China

**Keywords:** AKT signaling, apoptosis, ginsenoside Rk2, inflammation, ischemia–reperfusion, membrane translocation

## Abstract

Hepatic ischemia–reperfusion injury (IRI) poses a significant threat to clinical outcomes and graft survival during hemorrhagic shock, hepatic resection, and liver transplantation. Current pharmacological interventions for hepatic IRI are inadequate. In this study, we identified ginsenoside Rk2 (Rk2), a rare dehydroprotopanaxadiol saponin, as a promising agent against hepatic IRI through high‐throughput screening. The pharmacological effects and molecular mechanisms of Rk2 on hepatic IRI were further evaluated and elucidated in vitro and in vivo. Rk2 significantly reduced inflammation and apoptosis caused by oxygen‐glucose deprivation and reperfusion in hepatocytes and dose dependently protected against hepatic I/R‐induced liver injury in mice. Integrated approaches, including network pharmacology, molecular docking, transcriptome analysis, and isothermal titration calorimetry, along with experimental validation, indicated that Rk2 protects against hepatic IRI by targeting and activating the AKT (RAC serine/threonine protein kinase) signaling pathway. Pharmacological inhibition of AKT pathway or knockdown of AKT1 effectively diminished protective effects of Rk2. Rk2 directly binds to AKT1, facilitating its translocation from the cytoplasm to plasma membrane. This process markedly enhanced AKT interaction with PDPK1, promoting the activation of AKT1 and its downstream signaling. Our findings demonstrate that Rk2 protects against hepatic IRI by activating AKT signaling through direct binding to AKT1 and facilitating its membrane translocation.

## Graphical abstract

•Rk2, a rare dehydroprotopanaxadiol saponin, effectively alleviates hepatic IRI in vivo and in vitro.

•Rk2 represents a novel AKT1 agonist by facilitating its membrane translocation and phosphorylation.

•Rk2 enhances the interaction with PDPK1, leading to the activation of AKT1 and its downstream signaling.

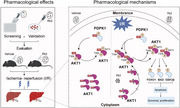

## 1 INTRODUCTION

Hepatic ischemia–reperfusion injury (IRI) is a significant complication that can occur during events such as hemorrhagic shock, extensive liver resection, and orthotopic liver transplantation. This condition can lead to severe liver damage, dysfunction, and even failure.^1^, ^2^ The progression of hepatic IRI involves a series of complex biological processes, including anaerobic metabolism, mitochondrial injury, oxidative stress, calcium overload, inflammatory responses, and various forms of cell death, such as apoptosis, autophagy, necrosis, and necroptosis.^2^, ^3^, ^4^, ^5^ Despite the introduction of numerous protective strategies, including ischemic pre‐conditioning, ischemic post‐conditioning, and mechanical reperfusion, the effectiveness of these interventions in relieving IRI remains limited.^6^, ^7^, ^8^ This underscores the need for ongoing research to better understand the underlying mechanisms of hepatic IRI and to develop more effective therapeutic approaches.

Recent advancements in understanding the molecular mechanisms underlying hepatic IRI and the identification of new biomarkers have opened up promising avenues for pharmacological treatments. Various intervention strategies have been developed to target key aspects of the injury process, including oxidative stress, metabolic modulation, inflammation, and cell death in hepatocytes.^5^, ^9^ In both animal models and clinical trials, several therapeutic agents have demonstrated efficacy in mitigating IRI.^5^, ^9^ These include calcium channel blockers (such as amlodipine), glucocorticoids, and antioxidants such as melatonin, ferritin, coenzyme Q10, and N‐acetyl cysteine. Additionally, polyethylene glycol has also shown potential in preserving liver function during IRI.^9^ Thus, the discovery and identification of pharmacological agents that can effectively prevent or minimize hepatic IRI have become a critical focus in the treatment of this condition.^5^, ^9^

In recent decades, there has been growing interest in the use of phytomedicines and natural products to treat various types of liver injury in preclinical studies.^10^, ^11^, ^12^, ^13^, ^14^ Ginsenosides, the main bioactive components of ginseng, have shown significant potential in protecting against multiple forms of IRI, including myocardial, cerebral, hepatic, renal, and intestinal IRIs. Their protective effects are attributed to several pharmacological activities, including antioxidant properties, free radical scavenging, autophagy inhibition, reduction of calcium overload, and anti‐inflammatory and anti‐apoptotic effects.^13^, ^16^, ^17^, ^18^, ^19^, ^20^ Notably, primary ginsenosides, such as Rg1 and Rb1, and total saponins from *Panax ginseng* and *Panax notoginseng*, have demonstrated effective liver protection against ischemic injury through the modulation of nuclear factor kappa‐B (NF‐κB) signaling pathway, CypD protein‐mediated mitochondrial apoptotic pathway, and the reactive oxygen species (ROS)‐nitric oxide (NO)‐hypoxia inducible factor (HIF)pathway.^15^, ^16^, ^17^ Collectively, these findings suggest that ginsenosides may may serve as promising protective agents for the treatment of hepatic IRI.

Rare ginsenosides (RGs), accounting for less than 0.1% of total extracts of the genus *Panax*, have garnered increasing attention due to their superior pharmacological activities compared to primary ginsenosides.^18^ RGs, such as ginsenoside Rk1, Rk2, Rg3, Rh2, and compound K, have been shown to protect against inflammation‐related liver diseases by reducing oxidative stress, inflammation, and apoptosis.^19^, ^20^, ^21^, ^22^, ^23^ However, the specific pharmacological effects of RGs on hepatic IRI have not been fully explored. Our preliminary data suggested that ginsenoside Rk2, a rare dehydroprotopanaxadiol saponin derived from steamed ginseng, has emerged as a promising candidate for treating hepatic IRI by high‐throughput screening in vitro. This study aims to further investigate the pharmacological effects of ginsenoside Rk2 on hepatic IRI and to elucidate the underlying molecular mechanism in vivo and in vitro.

## 2 RESULTS

### 2.1 Rk2 is a potential agent for the treatment of hepatic IRI

To gain insights into the pharmacological landscape and pharmacological potential of ginsenosides, we first conducted a network pharmacological analysis to identify potential targets and mechanisms associated with various ginsenosides documented in the Comparative Toxicogenomics Database (Figure S1A). Notably, the Disease Gene Network analysis suggested that ginsenosides may be beneficial for multiple pathological conditions, particularly metabolic diseases and reperfusion injury (Figure S1B). Consistent with these findings, Gene Ontology (GO) enrichment analyses revealed that ginsenosides are involved in biological processes related to IRI, including programmed cell death, apoptotic signaling pathways, and cellular responses to cytokines (Figure S1B). Furthermore, GO analysis of molecular functions indicated that target genes of ginsenosides were highly enriched for binding function (e.g., kinase, transcription factor, protein domain, protease, and ubiquitin protein ligase) and regulation of protein activity (e.g., signal receptor activator, enzyme activator, and nuclear receptor) (Figure S1C). Meanwhile, the Kyoto Encyclopedia of Genes and Genomes (KEGG) pathway analysis highlighted the connections between ginsenosides and liver injury‐related signaling pathways, including the cancer pathway, phosphatidylinositol 3‐kinase (PI3K)—AKT pathway, mitogen‐activated protein kinase pathway, Forkhead box O (FOXO) pathways, and autophagy (Figure S1D). However, a review of the literature indicated that the specific effects of ginsenosides on hepatic IRI are still not fully understood (Figure S1E).

**FIGURE 1:**
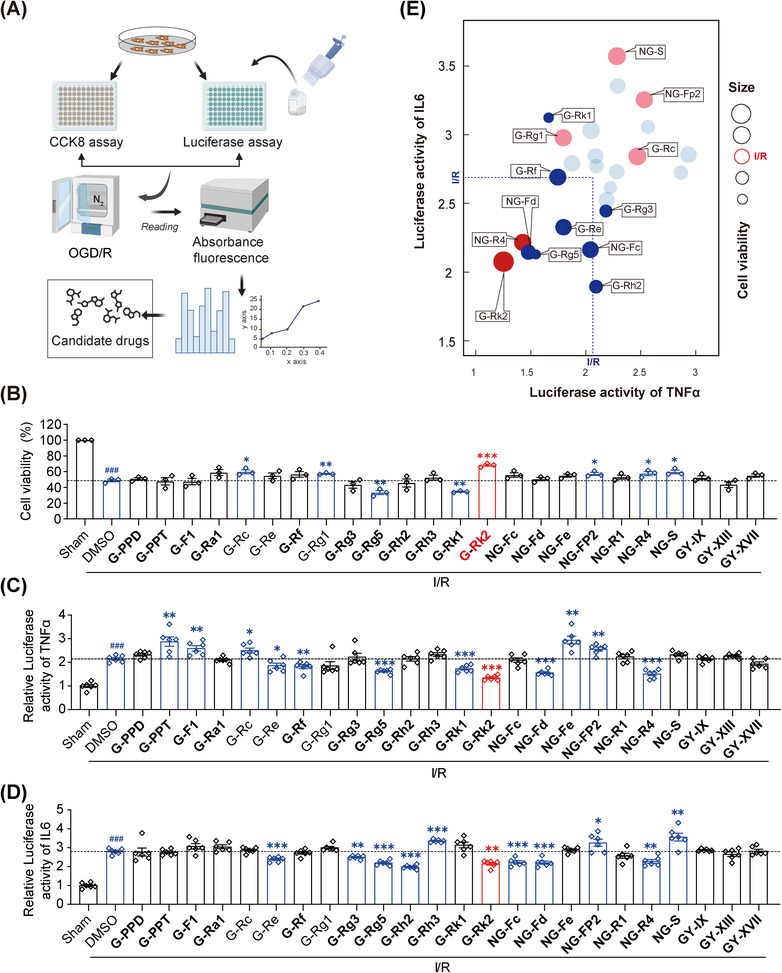
Ginsenoside Rk2 is a potential agent for the treatment of hepatic ischemia–reperfusion injury (IRI). (A) Graphical representation of the high‐throughput screening protocol for identifying effective agents in treating hepatic IRI from a set of ginsenosides. (B) Cell viability of HuH7 cells subjected to normal incubation or oxygen‐glucose deprivation and reperfusion (OGD/R) incubation after treatment with a set of ginsenosides at the concentration of 20 µM. (C) TNFα luciferase activity of HuH7 cells co‐transfected with the TNF‐α luciferase reporter plasmid and the Renilla luciferase reporter plasmid after OGD/R incubation and treatment with a set of ginsenosides at the concentration of 20 µM. (D) IL6 luciferase activity of HuH7 cells co‐transfected with the IL6 luciferase reporter plasmid and the Renilla luciferase plasmid after OGD/R incubation and treatment with a set of ginsenosides at the concentration of 20 µM. (**E**) Graphical representation of candidate agents for treating hepatic IRI based on the comprehensive analysis of cell viability as well as TNF‐α and IL6 luciferase activity. Blue: agents with anti‐inflammatory effects; pink or red: agents with increased cell viability; red: agents with anti‐inflammatory effects and increased cell viability. G: ginsenoside; NG: notoginsenoside; GY: gypenoside. All data are shown as the mean ± SEM of triplicate independent experiments. The font used for ginsenosides in bold denotes the rare types of ginsenosides. *p < 0.05, **p < 0.01, ***p < 0.001, versus DMSO‐treated group; ^###^ *p* < 0.001, vs. sham group.

To identify effective agents for hepatic IRI, we conducted a series of in vitro assays using a range of pharmacologically active ginsenosides (Table S1, Figure 1A). We assessed the cell viability of HuH7 cells under normal incubation conditions or after oxygen‐glucose deprivation and reperfusion (OGD/R) incubation, followed by exposure to either dimethyl sulfoxide (DMSO) or various ginsenosides at a concentration of 20 µM. As shown in Figure 1B, both primary ginsenosides (Rg1 and Rc) and RGs (Rk2, notoginsenoside Fp2, R4, and S) significantly improved cell viability of HuH7 cells and promoted their survival after OGD/R incubation. Additionally, primary ginsenoside Re and RG Rg5, Rk2, Fd, and R4 effectively reduced the luciferase activity of both TNF‐α and IL‐6 in HuH7 cells after OGD/R incubation, indicating their ability to inhibit IRI‐induced inflammatory responses (Figure 1C,D). Based on a comprehensive analysis of the high‐throughput screening results, which considered both cell viability and luciferase activities of TNFα and IL6, we identified ginsenoside Rk2 as an optimal agent for treating hepatic IRI (Figure 1E).

### 2.2 Rk2 inhibits inflammatory responses and hepatic apoptosis induced by OGD/R in vitro

**FIGURE 2:**
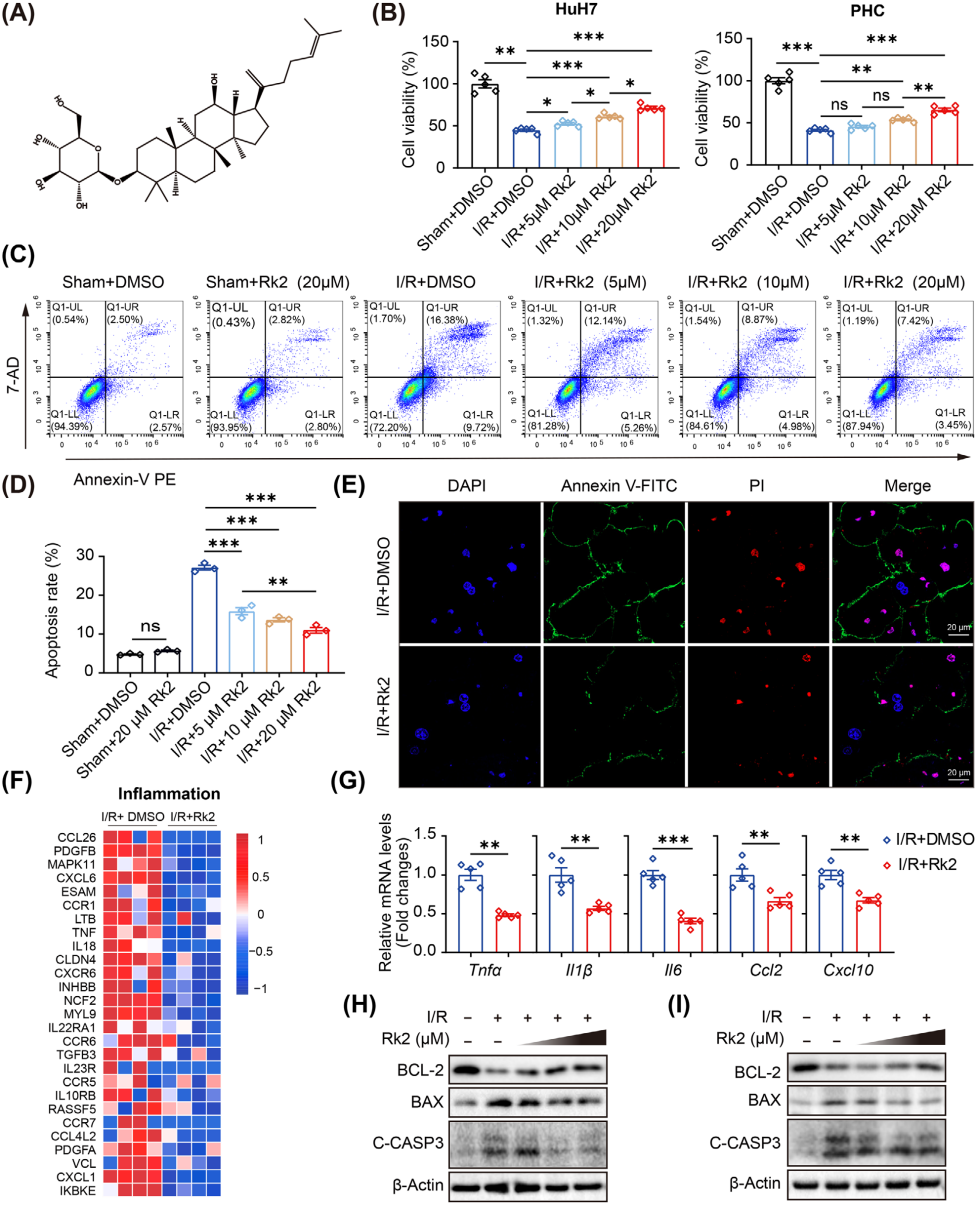
Rk2 inhibits inflammatory responses and hepatic apoptosis induced by oxygen‐glucose deprivation and reperfusion (OGD/R) **in vitro**. (A) Chemical structure of Rk2. (B) Cell viability of HuH7 cells and primary hepatocytes (PHCs) after OGD/R incubation and treatment with various concentrations of Rk2 (5 µM, 10 µM, and 20 µM). Each value is shown as the mean ± SEM of five replicates. (C and D) Flow cytometric analysis of apoptosis in HuH7 cells by staining with annexin V‐PE and 7‐AD. Graphic data for the apoptosis ratio are representative of three separate experiments. (E) Representative fluorescence microscopy images of annexin V‐FITC/PI staining for detecting apoptosis of PHCs after OGD/R incubation and treatment with Rk2 at the concentration of 20 µM. Cells with green (annexin V‐FITC) and red colors (PI) indicated that they were in the early apoptotic phase and late apoptotic phase, respectively. The fluorescence of green and red were employed to analyze the occurrence of early and late apoptosis, respectively. (F) The expression profile of genes related to the inflammatory response in HuH7 cells subjected to OGD/R incubation and treatment with or without Rk2 (20 µM) by RNA‐sequencing. Red, up‐regulated; blue, down‐regulated. (G) The mRNA levels of *Tnfa*, *Il1β*, *Il6*, *Ccl2*, and *Cxcl10* in PHCs after OGD/R incubation and treatments of Rk2 (20 µM). Each value is shown as the mean ± SEM of five replicates. (H and I) Western blot of BCL‐2, BAX, and cleaved CASPASE3 (c‐CASP3) protein levels in HuH7 cells (left) and PHCs (right) after OGD/R incubation and treatment with various concentrations of Rk2 (5 µM, 10 µM, and 20 µM). β‐Actin served as the internal control. All data represent three separate experiments, and values are shown as mean ± SEM. *p < 0.05, **p < 0.01, ***p < 0.001, n.s., not significant. PHCs, primary hepatocytes.

To establish the safe dosage range for subsequent in vitro experiments, we evaluated the cell viability of HuH7 cells and primary hepatocytes (PHCs) after 24 h of treatment with various concentrations of Rk2 under normal cell culture conditions. Rk2 demonstrated minimal toxicity at concentrations below 25 µM, while higher concentrations (above 50 µM) exhibited significant toxicity (Figure S2A). Next, HuH7 cells and PHCs were exposed to OGD for 6 h, followed by re‐oxygenation with medium containing normal glucose content and oxygen (20%) for an additional 6 h, creating an in vitro model of hepatic IRI. As shown in Figure 2B, OGD/R exposure led to a marked decrease in cell viability. However, treatment with Rk2 significantly mitigated this decline in a dose‐dependent manner. Flow cytometry analysis further demonstrated that Rk2 dose dependently reduced the apoptosis of HuH7 cells induced by OGD/R incubation (Figure 2C,D). Consistently, in PHCs, evaluation of apoptosis at both early and late stages using Annexin‐V and PI staining revealed that Rk2 at 20 µM effectively inhibited apoptosis, as indicated by a lower fluorescence intensity (Figure 2E and Figure S2B).

It is well recognized that inflammatory responses inflammatory responses contribute significantly to apoptosis induced by IRI.^24^ RNA‐sequencing (RNA‐seq)‐based heatmap analysis clearly showed that treatment with Rk2 markedly inhibited the expression of numerous genes associated with inflammatory responses and cell death in HuH7 cells after OGD/R incubation (Figure 2F and Figure S2C–E). Similarly, Rk2 notably decreased the mRNA levels of pro‐inflammatory genes, including tumor necrosis factor alpha (*Tnfα*), interleukin 1beta (*Il1β*), interleukin 6 (*Il6*), chemokine (CC‐motif), ligand 2 (*Ccl2*), and CXC chemokine ligand 10 (*Cxcl10*) in PHCs following OGD/R (Figure 2G). Additionally, Western blot assay indicated that Rk2 up‐regulated the expression of anti‐apoptotic factor B‐cell leukemia/lymphoma 2 (BCL‐2) in a dose‐dependent manner (Figure 2H,I). Concurrently, Rk2 down‐regulated the levels of the pro‐apoptotic factor BCL‐2‐associated X protein (BAX) and cleaved‐caspase3 (c‐CASP3) protein levels in both HuH7 cells and PHCs after OGD/R incubation (Figure 2H,I). This data strongly indicate that Rk2 effectively protects hepatocytes from inflammation and apoptosis induced by OGD/R in vitro.

### 2.3 Rk2 protects against hepatic I/R‐induced liver injury in mice

**FIGURE 3:**
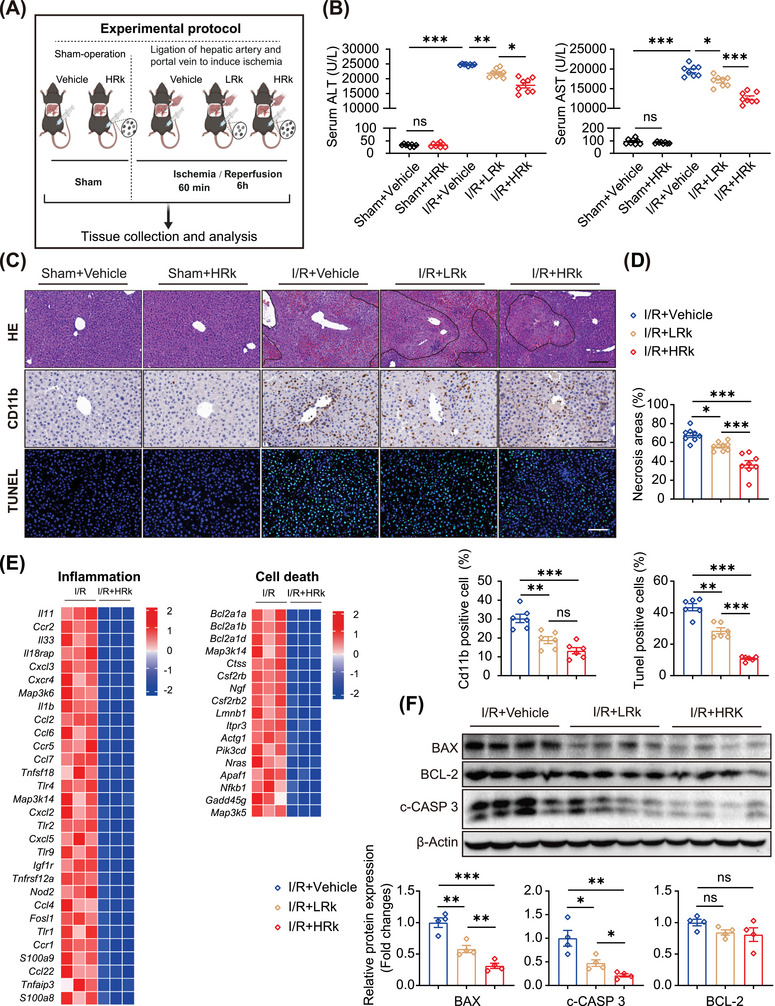
Rk2 protects against hepatic I/R‐induced liver injury in mice. (A) Experimental scheme of pharmacological treatment with Rk2 in mice. (B) Serum alanine aminotransferase (ALT) and aspartate aminotransferase (AST) levels, *n* = 8 mice per group. (C and D) Representative images of hematoxylin and eosin (H&E) staining (*n* = 8), immunohistochemistry for CD11b (*n* = 6), and TUNEL (*n* = 6) staining in liver sections from the mice (C) and their statistical charts (D). Values are shown as mean ± SEM. Scale bar = 100 µm. (E) Expression profile of genes related to inflammatory response and apoptosis between I/R group and I/R+HRk group by RNA‐sequencing. Red, up‐regulated; blue, down‐regulated. (F) Western blot (up) and the quantification (down) of protein levels of BCL‐2, BAX, and c‐CASP3 in liver tissue of mice. β‐Actin served as the internal control (*n* = 4). Values are shown as mean ± SEM. *p < 0.05, **p < 0.01, ***p < 0.001, n.s., not significant.

To assess the potential protective effects of Rk2 against hepatic IRI in vivo, mice underwent ligation of hepatic artery and portal vein, or a sham operation, with subsequent administration of low‐dose and high‐dose Rk2 prior to ischemia and at the beginning of reperfusion (Figure 3A). Hepatic IRI resulted in severe liver injury, characterized by dramatic increases in serum levels of alanine aminotransferase and aspartate aminotransferase (AST), extensive hepatocyte necrosis, and the prominent infiltration of CD11b‐positive cells and apoptotic cells in the ischemic area compared to the sham group (Figure 3B–D). However, these detrimental effects were alleviated in a dose‐dependent manner with the Rk2 pretreatment (Figure 3B–D). Additionally, we conducted an in vivo safety evaluation of Rk2 treatments, given the limited data on its effects in living organisms. As illustrated in Figure S3A,B, no adverse effects were observed following three consecutive days of Rk2 administration at a dose of 30 mg/kg (i.p.). The biochemical profiles of the liver, heart, and kidneys in Rk2‐treated mice remained within expected reference values compared to control animals. Histopathological evaluation using hematoxylin and eosin (H&E) staining of lung, heart, liver, spleen, and kidney revealed no significant difference between the Rk2‐treated and control groups (Figure 3C and Figure S3B). Furthermore, RNA‐seq was performed on liver samples from the I/R group and I/R+HRk group. Heatmaps and gene set enrichment analysis (GSEA) enrichment analysis indicated that Rk2 effectively suppressed the expression of numerous genes and multiple pathways associated with the inflammatory response and cell death (Figure 3E and Figure S3C–E).

Consistent with our in vitro findings, Rk2 administration dose dependently downregulated protein levels of the pro‐apoptotic regulators BAX and c‐CASP3 (Figure 3F). These data suggest that treatment with Rk2 effectively protects against IRI‐induced hepatic inflammation and damage in mice.

### 2.4 Rk2 targets and activates the AKT pathway during hepatic IRI

**FIGURE 4:**
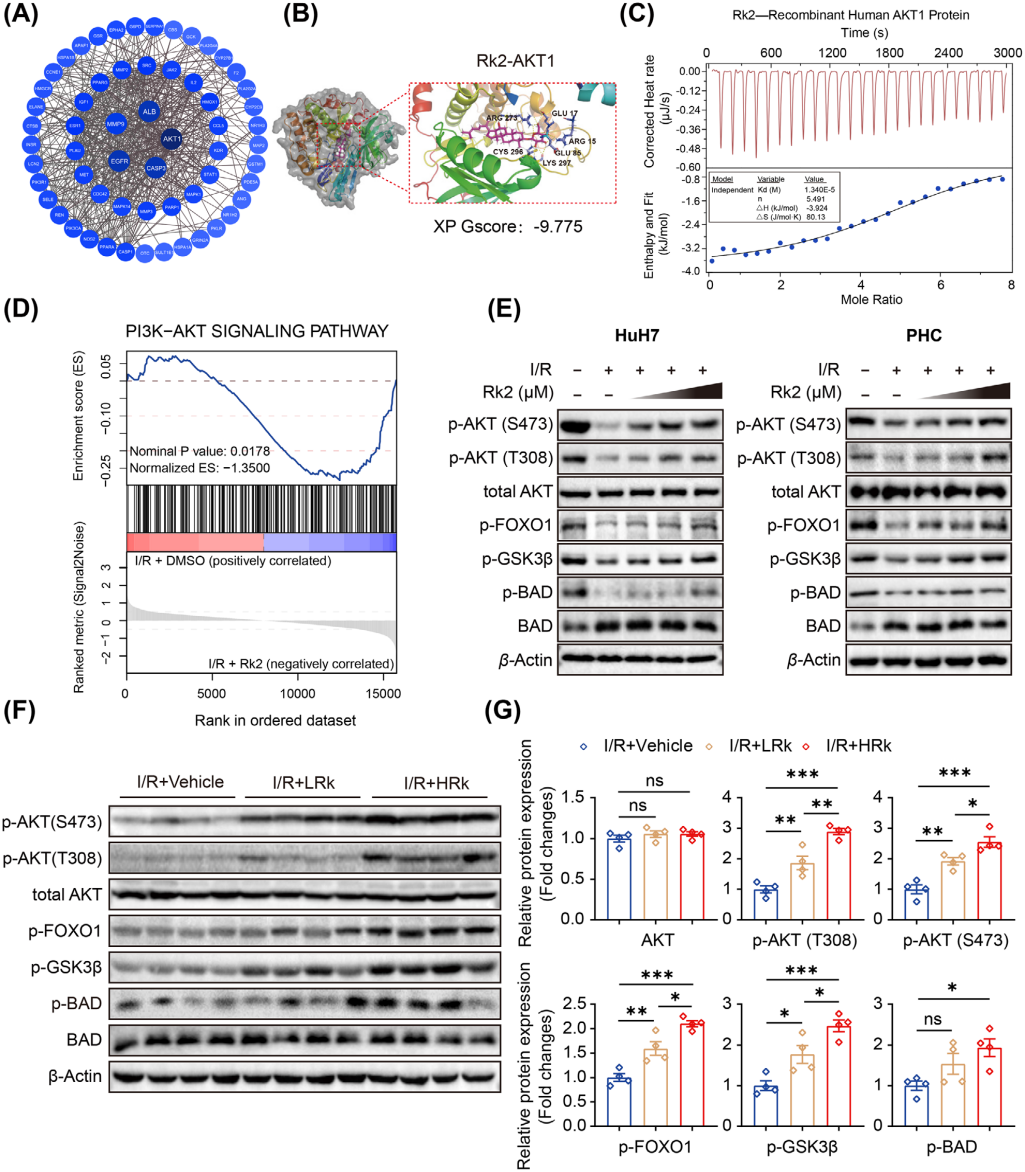
Rk2 targets and activates the AKT pathway during hepatic ischemia–reperfusion injury (IRI). (A) Protein–protein interaction (PPI) network constructed by the STRING database, highlighting the top five targets with the highest scores as key targets. (B) Molecular docking images and results showing the interaction between Rk2 and AKT1. (C) Thermodynamic analysis of the binding of Rk2 to human recombinant AKT1 protein at 25°C on a Nano ITC 200 instrument. (D) Gene set enrichment analysis (GSEA) results of activated PI3K‐AKT signaling pathway in HuH7 cells subjected to oxygen‐glucose deprivation and reperfusion (OGD/R) incubation and treatment with or without Rk2 (20 µM). (E) Protein expression of p‐AKT (T308 and Ser473), total AKT, p‐FOXO1, p‐GSK3β, p‐BAD, and BAD in HuH7 cells and primary hepatocytes (PHCs) under OGD/R incubation after treatment with various concentrations of Rk2 (5 µM, 10 µM, and 20 µM). β‐Actin serves as the internal control. The experiments were repeated independently three times with similar results. (F) Western blot and (G) their quantification of protein levels, including p‐AKT (T308 and S473), total AKT, p‐FOXO1, p‐GSK3β, p‐BAD, and BAD in liver tissue of mice. Values are shown as mean ± SEM (*n* = 4). *p < 0.05, **p < 0.01, ***p < 0.001, n.s., not significant. STRING, Search Tool for the Retrieval of Interacting Genes/Proteins; HIRI, hepatic ischemia/reperfusion‐injury.

To elucidate potential molecular mechanisms underlying the beneficial effects of Rk2 against hepatic IRI, we utilized target prediction information derived from the PharmMapper and chEMBL databases (Figure S4A). These databases identified 331 target candidates for Rk2 based on the chemical structure. Additionally, we collected 439 target proteins related to hepatic IRI from the DisGeNET and GeneCards databases. By comparing the predicted targets with hepatic IRI‐related proteins, we identified 60 overlapping genes, which are illustrated in the Venn diagram (Figure S4B). These 60 genes were then imported into the STRING database to construct a protein–protein interaction (PPI) network, comprising 60 protein nodes and 536 interaction edges. Topological analysis revealed the top five core proteins, including AKT1, ALB, CASP3, EGFR, and MMP9, based on their maximum clique centrality values (Figure 4A and Table S5).

To further investigate the binding affinity between Rk2 and these top proteins, the molecular docking analysis was conducted. A favorable and stable ligand‐receptor interaction is indicated by an XP Gscore below −6 and an MM‐GBSA dG Bind energy below −30 kcal/mol. The docking results revealed that Rk2 formed hydrogen bonds with residues Arg15, Glu17, Glu85, Arg273, Cys296, and Lys297 in AKT1, exhibiting the lowest XP Gscore (−9.775) and higher MM‐GBSA dG Bind energy (−26.35), compared to other predicted proteins (Figure 4B, Figure S4C,D, and Table S6). Additionally, thermodynamic analysis using isothermal titration calorimetry (ITC) confirmed that Rk2 directly binds to human recombinant AKT1 protein, with an exothermic reaction and moderate binding forces (Figure 4C). These data suggest that Rk2 likely targets AKT1 and exerts protective effects against hepatic IRI.

Clinical and experimental studies have indicated that manipulation and activating the AKT pathway can reduce hepatic damage during IRI.^25^, ^26^ Of note, the GSEA of transcriptome data from HuH7 cells indicated that Rk2 treatment resulted in the activation of the PI3K‐AKT signaling pathway in the context of OGD/R conditions (Figures 4C and S5A,B). Moreover, Rk2 has been shown to modulate a multitude of pathways and genes associated with the AKT1 signaling cascade, including the PI3K‐AKT signaling pathway, NF‐kappa B signaling pathway, and apoptosis pathway in murine models of hepatic IRI (Figures S3E and S5C). To gain further insight into the molecular mechanisms of Rk2, the effect of Rk2 treatment on the AKT pathway during hepatic IRI was investigated. Western blot analysis showed that Rk2 treatment dose dependently increased the phosphorylation levels of AKT at Thr308 and Ser473 in HuH7 cells and PHCs after OGD/R. This activation subsequently led to the activation of downstream target proteins, including FOXO1, glycogen synthase kinase 3 beta (GSK3β), and BCL2‐associated agonist of cell death (BAD) in vitro (Figure 4E). Moreover, Rk2 administration in mice promoted the dose‐dependent activation of AKT phosphorylation and its downstream signaling pathways in the liver, as evidenced by increased levels of phosphorylated AKT (p‐AKT) at both Thr308 and Ser473, as well as phosphorylated FOXO1, GSK3β, and BAD in the context of hepatic IRI (Figure 4F,G).

### 2.5 Inhibition of PI3K/AKT signaling reverses the protective effects of Rk2 in hepatocytes

**FIGURE 5:**
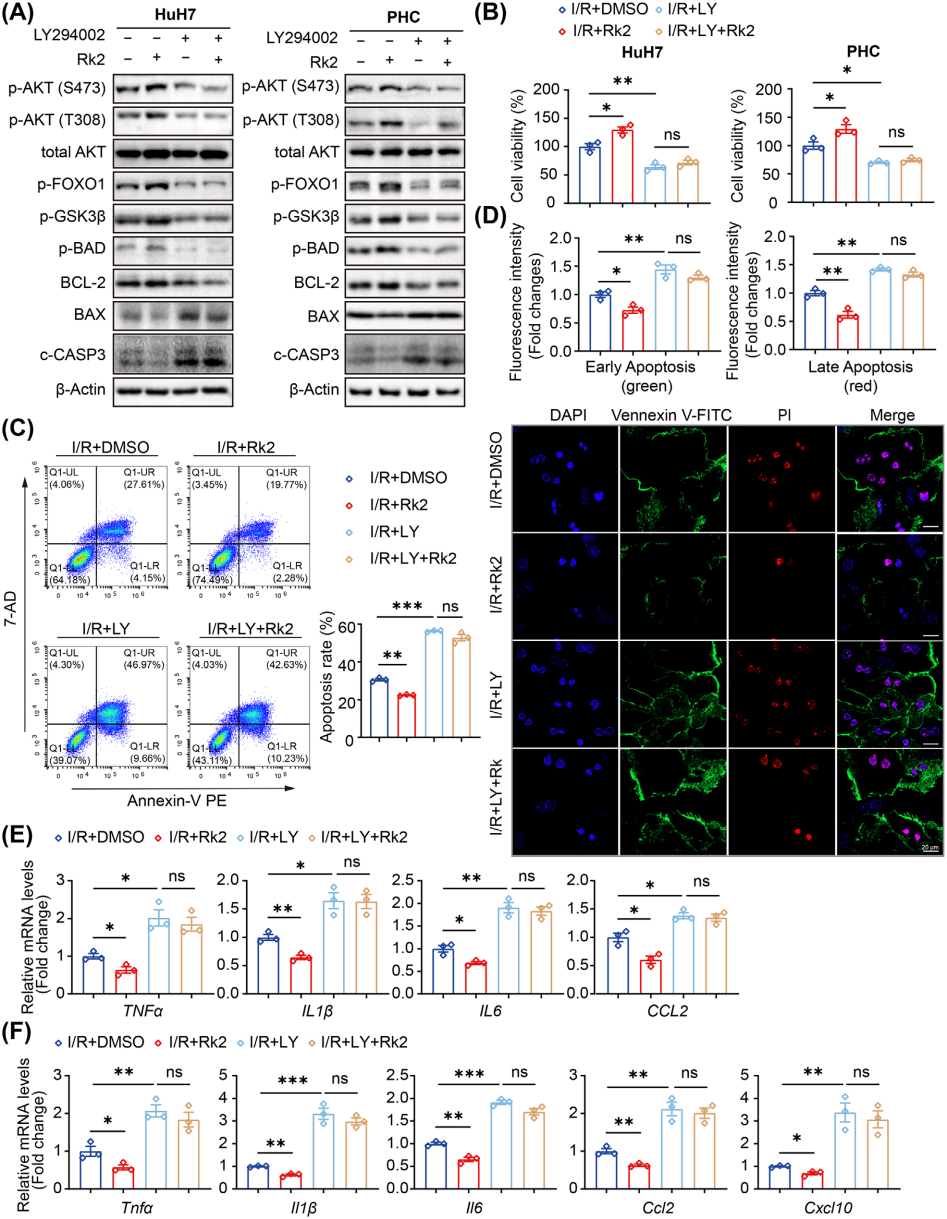
Inhibition of PI3K‐AKT signaling reverses the protective effects of Rk2 in hepatocytes. (A) Western blot analysis of protein levels, including p‐AKT, total AKT, p‐FOXO1, p‐GSK3β, p‐BAD, BCL‐2, BAX, and c‐CASP 3 in HuH7 cells and PHCs subjected to oxygen‐glucose deprivation and reperfusion (OGD/R) incubation after treatment with Rk2 (20 µM) in the presence or absence of LY294002 (30 µM). Bands are representative of three separate experiments. (B) Cell viability of HuH7 cells and primary hepatocytes (PHCs) subjected to OGD/R incubation after treatment with Rk2 (20 µM) in the presence or absence of LY294002 (30 µM). Values are shown as the mean ± SEM of three replicates. (C) Flow cytometric analysis of apoptosis of HuH7 cells with OGD/R incubation by staining with annexin V‐PE and 7‐AD after treatments with Rk2 (20 µM) in the presence or absence of LY294002 (30 µM). (D) Representative fluorescence microscopy images (down) of annexin V‐FITC/PI staining and quantitative results (up) of early and late apoptosis based on the fluorescence intensity of green and red in PHCs following OGD/R incubation and treatments with Rk2 (20 µM) in the presence or absence of LY294002 (30 µM). Scale bar = 20 µm. (**E**) qPCR analysis of *TNFα*, *IL1β*, *IL6*, and *CCL2* in HuH7 cells. (**F**) qPCR analysis of *Tnfa*, *Il1b*, *Il6*, *Ccl2*, and *Cxcl10* in PHCs. Data are shown as the mean ± SEM of three replicates. *p < 0.05, **p < 0.01, ***p < 0.001, n.s., not significant.

To investigate the role of AKT activation for the protective effects of Rk2 against hepatic IRI, LY294002 (LY), a PI3K/AKT signaling inhibitor was used to treat HuH7 cells and PHCs before OGD/R in the presence or absence of Rk2. The results demonstrated that the pre‐treatment of LY effectively inhibited the levels of p‐AKT at Thr308 and Ser473, as well as the activation of AKT substrate proteins, such as FOXO1, GSK3β, and BAD. Consequently, LY up‐regulated the expression of pro‐apoptotic proteins (BAX and c‐CASP3) in both HuH7 cells and PHCs following OGD/R, compared to the I/R group. Importantly, when HuH7 cells and PHCs were pretreated with LY294002, treatment with Rk2 at a concentration of 20 µM failed to activate AKT and its downstream target proteins (Figure 5A). Furthermore, LY pre‐treatment led to a decrease in cell viability in both HuH7 cells and PHCs after reperfusion, reversing the increased cell viability observed with Rk2 treatment (Figure 5B). Similarly, LY also abolished the anti‐inflammatory and anti‐apoptotic effects of Rk2 in HuH7 cells and PHCs subjected to OGD/R (Figure 5C–F). These results indicate that inhibition of AKT signaling negates the protective effects of Rk2 against hepatic IRI in vitro.

### 2.6 Endogenous AKT1 interruption abolishes the beneficial role of Rk2 in hepatocytes

**FIGURE 6:**
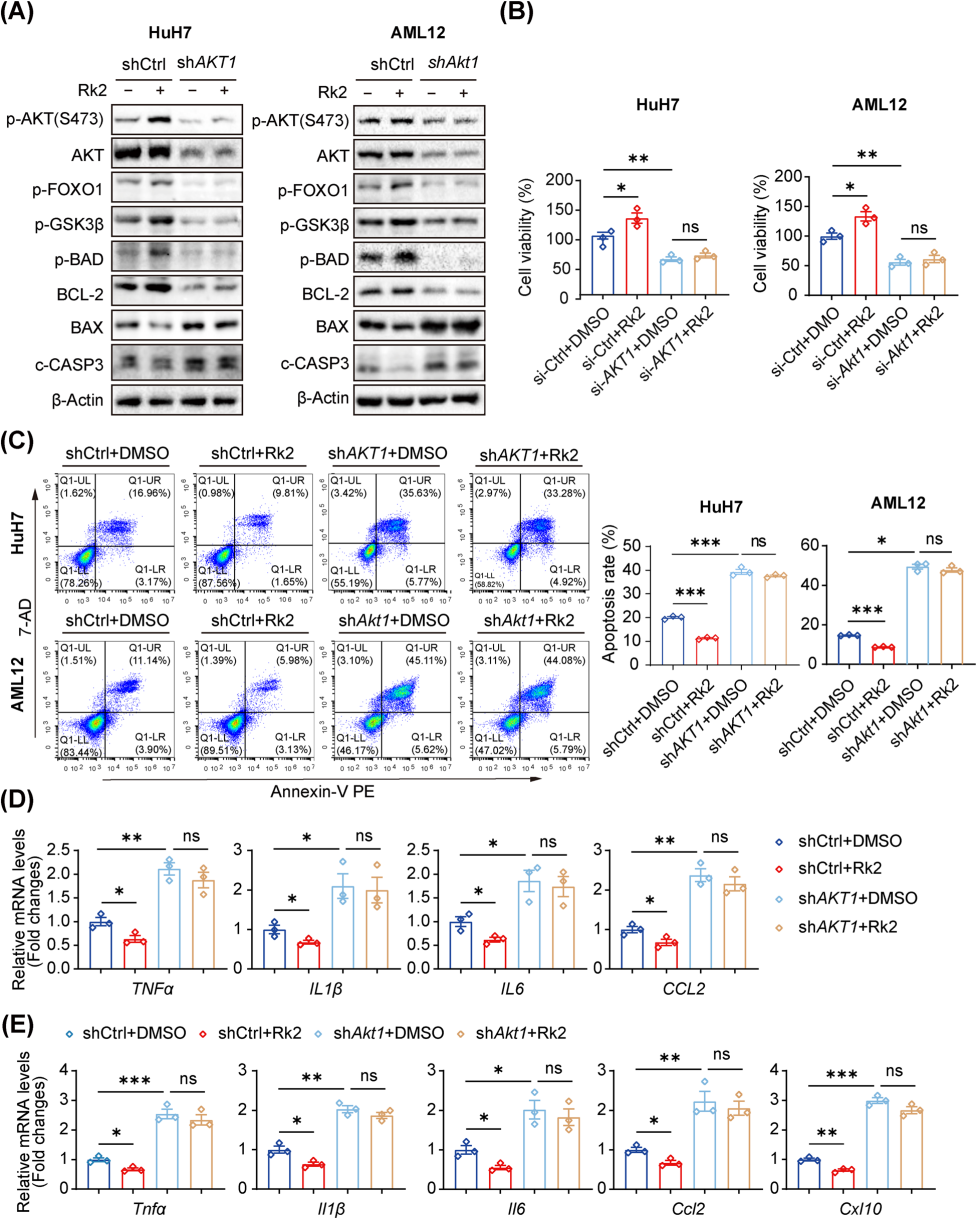
Endogenous AKT1 interruption abolishes the beneficial role of Rk2 in hepatocytes. (A) Protein expression of p‐AKT, total AKT, p‐FOXO1, p‐GSK3β, p‐BAD, BCL‐2, BAX, and c‐CASP 3 in HuH7 and AML12 cells with endogenous AKT1 knockdown after oxygen‐glucose deprivation and reperfusion (OGD/R) incubation and treatment with Rk2 (20 µM). (B) Cell viability of HuH7 and AML12 cells with endogenous AKT1 knockdown after OGD/R incubation and treatment with Rk2 (20 µM). (C) Flow cytometric analysis of apoptosis in HuH7 and AML12 cells with endogenous AKT1 knockdown, assessed by staining with annexin V‐PE and 7‐AD after OGD/R incubation and treatments with Rk2 (20 µM). (D) qPCR analysis of *TNFα*, *IL1β*, *IL6*, and *CCL2* in HuH7 cells with endogenous *AKT1* knockdown. (E) qPCR analysis of *Tnfa*, *Il1b*, *Il6*, *Ccl2*, and *Cxcl10* in AML12 cells with endogenous *Akt1* knockdown. Data are shown as the mean ± SEM of three replicates. *p < 0.05, **p < 0.01, ***p < 0.001, n.s., not significant.

The AKT kinase family comprises three highly homologous isoforms: AKT1, AKT2, and AKT3, with AKT1 being primarily essential for cell survival.^27^ In this study, we constructed cell lines with stable knockdown of *AKT1* in HuH7 and AML12 cells using RNA interference to further assess the pharmacological role of Rk2 in vitro during hepatic IRI. Consistent with the effects of pharmacological AKT inhibition, the knockdown of endogenous AKT1 in HuH7 cells and AML12 cells resulted in reduced AKT1 protein levels and inhibited downstream signaling pathways, which was evidenced by decreased levels of p‐AKT, phosphorylated FOXO1 (p‐FOXO1), phosphorylated GSK3β (p‐GSK3β), and phosphorylated BAD (p‐BAD), alongside increased levels of BAX and c‐CASP3 (Figure 6A). These alterations ultimately led to decreased cell viability, exacerbated inflammation responses, and increased apoptosis (Figure 6B–E). In contrast, treatment with Rk2 significantly enhanced cell viability and inhibited inflammation responses and hepatic apoptosis by activating the AKT pathway and its downstream signaling molecules, compared with the shCtrl group (Figure 6B–E). Notably, the beneficial effects of Rk2 treatment during IRI were diminished when AKT1 was knocked down in hepatocytes, showing similar outcomes between the sh*AKT1* and sh*AKT1*+Rk2 groups. These data suggest that the protective effects of Rk2 were largely attenuated in HuH7 and AML12 cells after the knockdown of endogenous AKT1.

### 2.7 Pharmacological inhibition of Akt signaling diminishes the protective role of Rk2 in hepatic IRI in mice

**FIGURE 7:**
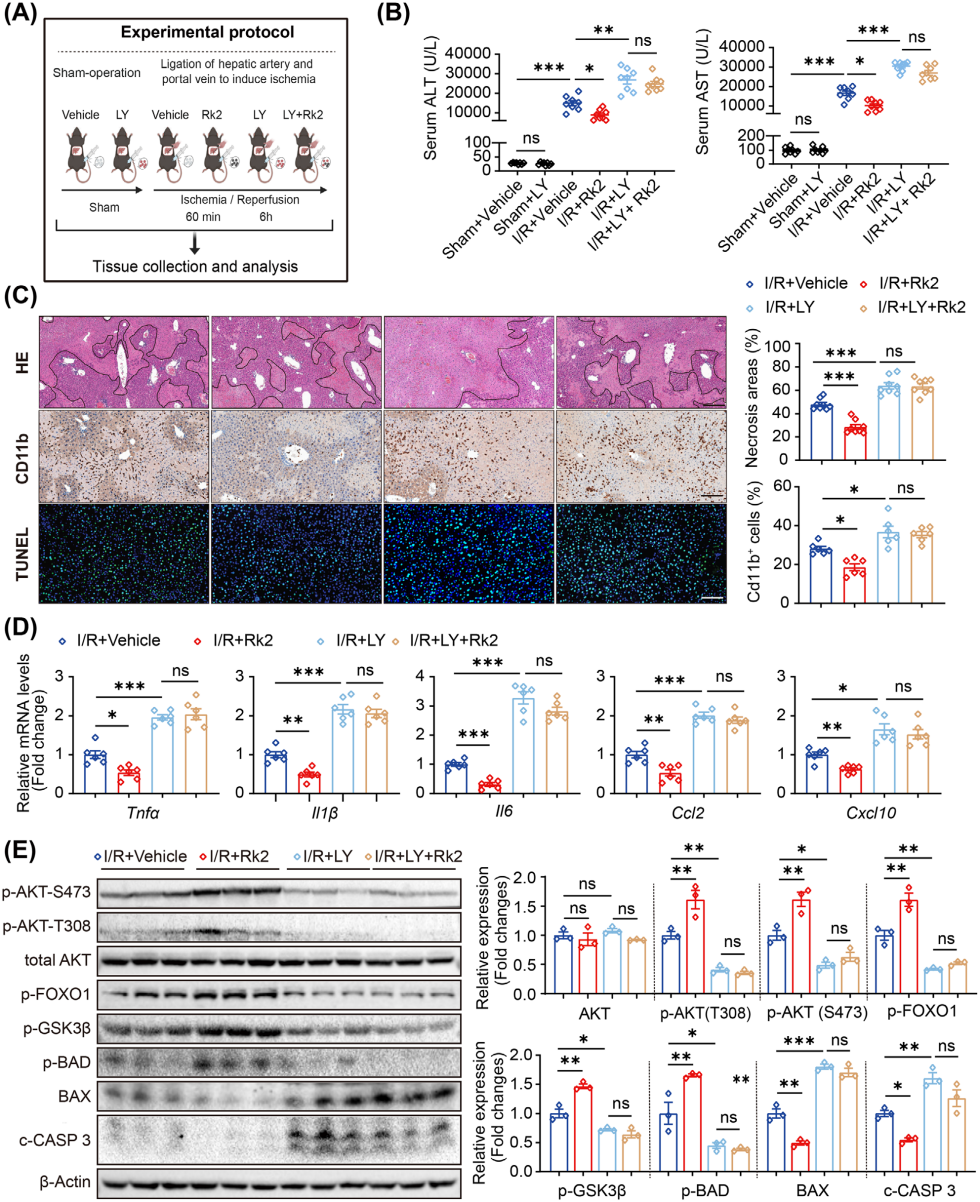
Pharmacological inhibition of Akt signaling diminishes the protective role of Rk2 in hepatic ischemia–reperfusion injury (IRI) **in mice**. (A) Scheme of the rescue experiment in mice. (B) Serum alanine aminotransferase (ALT) and aspartate aminotransferase (AST) levels of mice (*n* = 8 mice per group). (C) Representative images of hematoxylin and eosin (H&E) staining (*n* = 8), immunohistochemistry for CD11b (*n* = 6) and TUNEL (*n* = 6) in liver sections of mice (left, scale bar = 100 µm), and their statistical charts (right). (D) The mRNA levels of *Tnfa*, *Il1b*, *Il6*, *Ccl2*, and *Cxcl10* of liver tissue in mice, *n* = 6. (E) Western blot analysis (left) and the quantification (right) of protein levels of p‐AKT, total AKT, p‐FOXO1, p‐GSK3β, p‐BAD, BAX, and c‐CASP3 in liver tissue of mice. Values are shown as mean ± SEM. *p < 0.05, **p < 0.01, ***p < 0.001, n.s., not significant. LY, LY294002.

To verify the protective role of activated AKT signaling by Rk2 in mice with hepatic IRI, LY294002 was administered intraperitoneally to mice before ischemia and at the beginning of reperfusion (Figure 7A). LY administration exacerbated hepatic IRI in mice, as indicated by elevated serum transaminase levels, increased necrotic areas, severer infiltration of CD11b‐positive cells, and higher number of apoptotic cells in the ischemic area compared with vehicle treatment (Figure 7B,C and Figure S6). Treatment with Rk2 effectively alleviated hepatic IRI in mice. However, co‐administration of LY and Rk2 diminished the hepatoprotective effects of Rk2, resulting in phenotypes similar to those observed in LY alone (Figure 7B,C and Figure S6).

Furthermore, LY treatment intensified hepatic inflammatory responses, as evidenced by increased mRNA levels of pro‐inflammatory genes, such as *Tnfa, Il1β, Il6, Ccl2*, and *Cxcl10*, and compromised the anti‐inflammatory effects of ginsenoside Rk2 in vivo (Figure 7D). Additionally, LY treatment led to decreased AKT phosphorylation and subsequent activation of downstream effector proteins, resulting in an increase in pro‐apoptotic protein expression and a decrease in anti‐apoptotic protein expression, effectively neutralizing the benefits of Rk2 (Figure 7E). Consequently, Rk2 failed to exhibit hepatic protective effects in the liver when the AKT signaling pathway was inhibited by LY. These results indicate that pharmacological inhibition of AKT signaling significantly diminishes the protective effects of Rk2 against hepatic IRI in mice.

### 2.8 Rk2 facilitates AKT activation by enhancing its translocation from the cytoplasm to the plasma membrane

**FIGURE 8:**
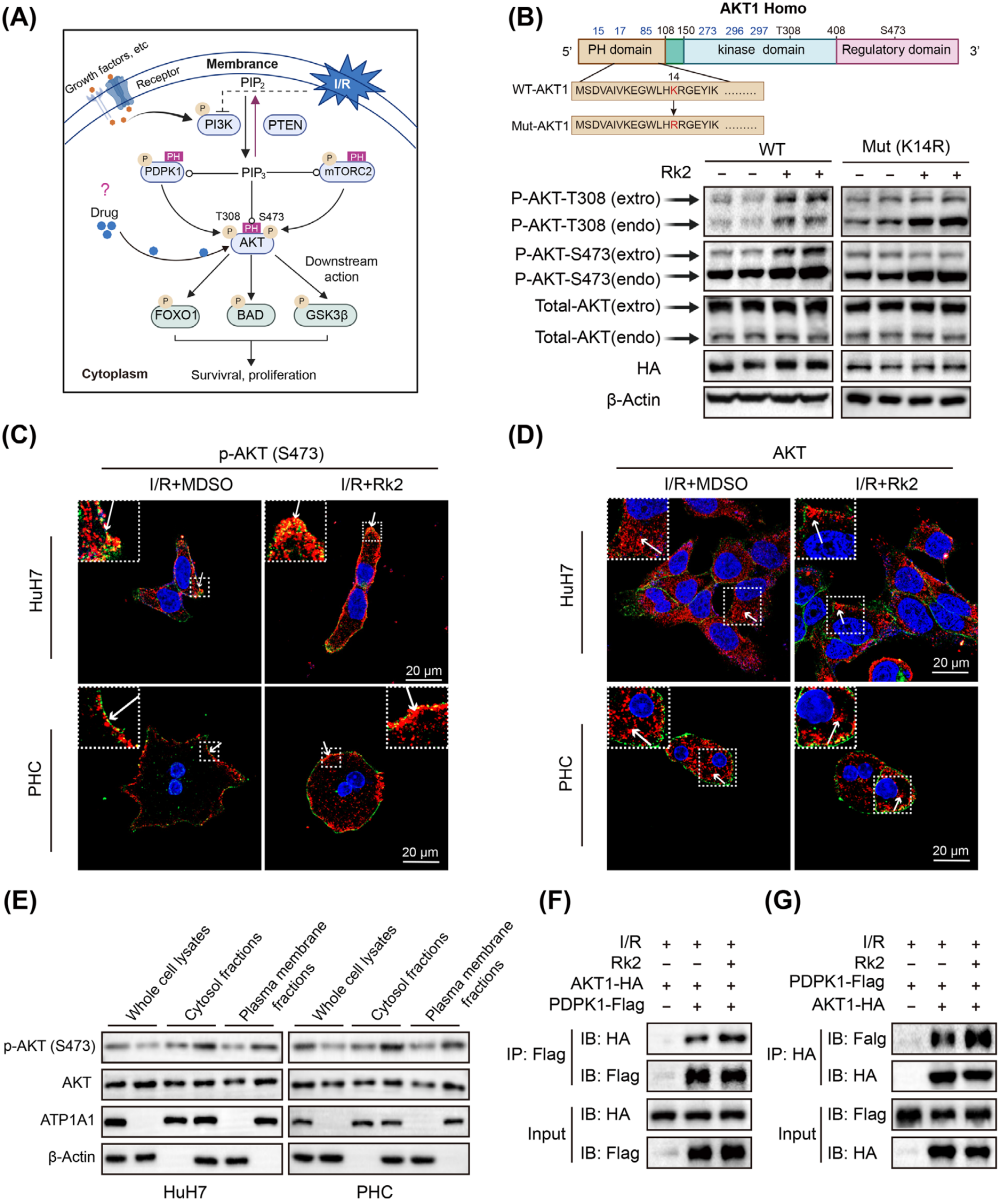
Rk2 facilitates AKT activation by enhancing its translocation from the cytoplasm to the plasma membrane. (A) Schematic illustration of canonical mechanisms of AKT activation. (B) Schematic domain structure of the human AKT1 protein and a mutant variant of its PH domain (up), and western blot analysis (down) of protein levels of endogenous and exogenous p‐AKT (Thr308 and Ser473) in two types of cell lines stably expressing exogenous wild cherry HA‐tagged AKT1 and cherry HA‐tagged AKT (K14R) mutant after oxygen‐glucose deprivation and reperfusion (OGD/R) incubation and treatment with Rk2 (20 µM). The amino acid position labeled in blue are Rk2 binding sites. (C) Representative fluorescence microscopy images of expression and subcellular localization of endogenous p‐AKT (Ser473) in primary hepatocytes (PHCs) and HuH7 cells after OGD/R incubation and treatment with Rk2 (20 µM). (D) Representative fluorescence microscopy images of expression and subcellular localization of endogenous AKT in PHCs and HuH7 cells after OGD/R incubation and treatment with Rk2 (20 µM). (E) The protein expression of p‐AKT (Ser473) and total AKT in whole cell lysates, cytosol fractions, and plasma membrane fractions in HuH7 cells (left)/PHCs (right) after OGD/R incubation and treatment with Rk2 (20 µM). β‐Actin and ATP1A1 were used as internal controls for cytoplasmic proteins and membrane proteins, respectively. Bands are representative of three separate experiments. (F) Co‐immunoprecipitation of HA‐tagged AKT1 and Flag‐tagged PDPK1 detected by western blot using anti‐Flag antibodies. Vectors expressing HA‐tagged AKT1 or Flag‐tagged PDPK1 were transiently co‐expressed in HuH7 cells with OGD/R incubation and treatment with Rk2 (20 µM). (G) Co‐immunoprecipitation of HA‐tagged AKT1 and Flag‐tagged PDPK1 detected by western blot using anti‐HA antibodies. Vectors expressing HA‐tagged AKT1 or Flag‐tagged PDPK1 were transiently co‐expressed in HuH7 cells with OGD/R incubation and treatment with Rk2 (20 µM).

To better understand the molecular mechanism by which Rk2 activates AKT signaling, we first examined the expression of AKT upstream mediators following Rk2 treatment under I/R conditions (Figure 8A). Notably, Rk2 did not affect the activation of upstream kinases, such as PI3K and 3‐phosphoinositide‐dependent protein kinase 1 (PDPK1), nor did it impact the phosphatase and tensin homolog (PTEN), which negatively regulates the AKT signaling pathway (Figure S7A–C). Additionally, the knockdown of *RICTOR*, a critical subunit of mammalian target of rapamycin complex 2 (mTORC2) that phosphorylates AKT at Ser473, did not significantly impair the function of Rk2 on AKT activation (Figure S7D,E).

Beyond regulating upstream components, the translocation of AKT from the cytoplasm to the plasma membrane, mediated by phosphatidylinositol (3,4,5) trisphosphate (PIP_3_), is a crucial step for AKT activation.^28^ To investigate whether Rk2 facilitates this membrane translocation of AKT during IRI, we generated two types of HuH7 cell lines: one expressing exogenous wild‐type cherry‐HA‐tagged AKT1 and the other expressing cherry‐HA‐tagged AKT (K14R) mutant that lacks the PIP_3_ binding capability.^29^ After subjecting these cell lines to OGD and Rk2 treatment, we observed that Rk2 enhanced the phosphorylation of both endogenous and exogenous AKT1 at Thr308 and Ser473 in the wild‐type cells by binding to the pleckstrin homology (PH) and kinase domains of AKT1 (Figure 8B). However, Rk2 failed to activate the mutant AKT (K14R), despite the increased phosphorylation of endogenous AKT1 being observed in the mutant cell lines (Figure 8B). Consistent with these results, Rk2 treatment resulted in a greater presence of wild‐type AKT1 near the plasma membrane, while the AKT (K14R) mutant predominantly remained in the cytoplasm after Rk2 incubation (Figure S7F). Additionally, Rk2 increased the expression of phosphorylated AKT (Ser473) in both PHCs and wild‐type HuH7 cells after OGD/R, with most of the elevated phosphorylated AKT (Ser473) localized at plasma membrane (Figure 8C and Figure S7G,H). Rk2 also upregulated the expression of phosphorylated AKT (Ser473) at plasma membrane in HuH7 cells upon insulin stimulation (Figure S7I). These results indicated that the activation of AKT by Rk2 is dependent on PIP_3_‐mediated membrane recruitment and Rk2 probably modulates the membrane translocation of AKT1. To further explore the relationship between Rk2‐mediated AKT activation and membrane translocation, we conducted a comprehensive examination of AKT and phosphorylated AKT (Ser473) levels in whole‐cell lysates, cytosol, and plasma membrane fractions. Immunofluorescence and western blot assays demonstrated an increased level of AKT1 at the plasma membrane and within the plasma membrane fraction in both PHCs and HuH7 cells, compared to the vehicle group (Figure 8D,E, Figures S7J,K and S8). Moreover, co‐immunoprecipitation assays indicated that Rk2 enhanced the interaction between AKT1 and its upstream target, PDPK1, primarily due to the increased presence of AKT1 at the plasma membrane (Figure 8F,G). These findings suggest that Rk2 directly binds to AKT1 at both the PH and the kinase domains and facilitates its translocation from the cytoplasm to plasma membrane for further activation under I/R conditions.

## 3 DISCUSSION

Hepatic IRI is a significant concern in hemorrhagic shock, hepatic resection, and liver transplantation, as it can adversely affect clinical outcomes and graft survival.^1^, ^2^ Consequently, it is an urgent need to investigate potential therapeutic interventions that can mitigate hepatic IRI and enhance patient outcomes. In recent years, extensive evidence and clinical data have emerged indicating that *Panax ginseng*, *Panax notoginseng*, and their active constituents, known as ginsenosides, have been extensively studied for their efficacy in treating myocardial IRI and cerebral IRI.^30^, ^31^, ^32^, ^33^ Despite the promising findings related to ginsenosides in other contexts, there is a notable lack of research specifically exploring their potential in the treatment of hepatic IRI. In this study, we identified an RG, designated Rk2, as a potential protective agent for hepatic IRI through multiple screening assays. Our data indicated that Rk2 pretreatment effectively attenuated the hepatic damage and apoptosis induced by IRI. In addition to Rk2, several other RGs, such as ginsenoside Rg5, notoginsenoside Fc, Fd, and R4, demonstrated superior potential against hepatic IRI in vitro compared to primary ginsenosides, particularly in terms of their anti‐inflammatory effects. The enhanced pharmacological effects of RGs may be attributable to differences in their chemical structures, including variations in the number of sugar molecules, sugar linkages, and the localization of double‐bond positions.^18^ It is hypothesized that the enhanced biological activity of Rk2 may be attributed to its unique chemical structure, which comprises a sugar molecule and a double bond at positions C‐20 and C‐21.

Hepatic IRI encompasses a series of complex events, including inflammatory responses and apoptosis, which significantly contribute to hepatocyte damage and liver injury.^34^, ^35^ Consequently, targeting signaling pathways associated with inflammation and apoptosis has emerged as a promising therapeutic strategy to mitigate hepatic IRI and improve cell function and survival. In recent decades, numerous clinical and experimental studies have highlighted the role of the serine/threonine kinase AKT as a crucial target in IRI.^25^, ^26^ AKT regulates multiple downstream mediators that influence cellular survival, death, and proliferation, including FOXO transcription factors, GSK3β, and BAD.^36^, ^37^, ^38^ Activation of the AKT pathway through ischemic preconditioning, postconditioning, or pharmacological agents has been shown to alleviate hepatic IRI in both clinical and experimental settings.^25^, ^38^, ^39^, ^40^ In this study, we demonstrated that Rk2 effectively reduced hepatic inflammation and apoptosis during hepatic IRI by modulating the activation of AKT1, which indicates that Rk2 influences the AKT1 signaling pathway, ameliorating inflammatory responses and apoptosis in the liver.

The activation of AKT1 follows a well‐defined canonical pathway. In its inactive form, AKT1 is translocated from the cytoplasm to the plasma membrane in a PIP_3_‐dependent manner. This translocation is subsequently followed by the phosphorylation of two critical residues: Thr308 in the catalytic domain and Ser473 in the C‐terminal hydrophobic motif. While, phosphorylation at Thr308 partially activates AKT signaling, it is necessary for initiating this process, full activation requires subsequent phosphorylation at Ser473.^41^ Multiple upstream components, including PI3K, PDPK1, mTORC2, and PTEN, play essential roles in initiating and regulating this pathway.^28^, ^42^, ^43^ The Class I PI3K family generates PIP_3_ from PI(4,5)P_2_ at the plasma membrane, facilitating the recruitment of AKT1, PDPK1, and mTORC2 through the binding of their PH domains, which induces conformational changes.^28^, ^44^ This recruitment allows for the phosphorylation of AKT1's activation loop (Ser473) and hydrophobic motif (Thr308) by PDK1 and mTORC2, respectively, at the plasma membrane. Concurrently, the phosphatase PTEN can terminate the activating signal by dephosphorylating PIP_3_, reverting it back to PI(4,5)P_2_.^28^, ^42^ Conversely, in the absence of PIP_3_ or mutations in the PH domain, AKT undergoes a rapid dephosphorylation as a consequence of an autoinhibitory process driven by the interaction between its PH and kinase domains.^44^ Notably, our findings indicate that Rk2 activates AKT1 primarily through PIP_3_ recruitment and subsequent membrane relocalization, without significantly impacting the functional activation of upstream components. Moreover, Rk2 directly binds to AKT1 at both the PH and kinase domains, facilitating its intracellular trafficking with conformational changes. This process leads to increased levels of AKT1 at the plasma membrane upon PIP_3_ stimulation. Resultantly, Rk2 further enhances the interaction between AKT1 and PDPK1, thereby facilitating the initiation of AKT1 phosphorylation at Thr308 and subsequent full phosphorylation at Ser473, which ultimately activates AKT1 and its downstream effects under I/R conditions. This functional activation may be predominantly attributed to conformational changes induced by Rk2 binding, promoting the membrane recruitment with disruption of the inhibitory PH–kinase interface.^44^, ^45^

In addition to the PIP_3_‐dependent activation, there are reports of allosteric activation of cytosolic AKT through chemical agents that bypass the need for membrane relocalization.^29^ For instance, SC79, a specific AKT activator, has been shown to induce cytosolic activation of AKT independent of PIP_3_‐mediated membrane translocation, exerting protective effects in hepatic IRI.^29^, ^39^ Our findings suggest that Rk2 may serve as an alternative AKT activator that modulates the AKT1 relocalization without directly influencing upstream kinases and regulators. Other ginsenosides, such as Rb1, Rg5, and compound K, have also been reported to alleviate ischemia–reperfusion injury in myocardial and intestinal tissues through the activation of AKT signaling.^46^, ^47^, ^48^, ^49^ These effects probably stem from the structured biological activity of ginsenosides.

In recent years, several pharmacological agents targeting the AKT pathway have been investigated as potential treatments for hepatic IRI.^17^, ^39^ However, many of these studies have not clearly elucidated the specific targets and mechanisms involved in the AKT pathway. Our study aims to explore the exact mechanism underlying the protective benefits of Rk2 in hepatic IRI. Moreover, Rk2 has demonstrated the anti‐inflammatory, anti‐apoptotic, and anti‐oxidative properties in various conditions, such as ulcerative colitis^21^ and alcoholic liver disease,^19^ by regulating the ERK/MEK pathway and NLRP inflammasome pathways. Our findings contribute to the growing body of pharmacological research on Rk2 and its potential role in treating hepatic IRI. In this study, we employed an in vitro approach to screen and identify ginsenosides with potential therapeutic effects on hepatic IRI. However, this methodology has inherent limitations, including potential selection bias in the evaluation indicators and the specific hepatocyte I/R models utilized. Consequently, the potential benefits of Rk2 may have been underestimated, particularly beyond its anti‐inflammatory and anti‐apoptotic effects. Given the likelihood that Rk2 possesses a diverse range of biologically active properties, further investigation into its comprehensive therapeutic potential is warranted. Additionally, technical challenges related to the labeling or modification of Rk2 for direct identification of protein targets constrain our findings. Considering the complexity of potential targets and associated mechanisms, further research is essential to elucidate the underlying mechanisms of action and molecular targets of Rk2.

In summary, our data provide compelling evidence that ginsenoside Rk2 exerts protective effects against hepatic IRI through promoting the activation of AKT signaling. By directly binding to AKT1 and facilitating its membrane translocation, Rk2 effectively mitigates hepatic inflammation and apoptosis. These findings illuminate the protective potential of ginsenoside Rk2 and enhance our understanding of the underlying mechanisms involved in hepatic IRI.

## 4 MATERIALS AND METHODS

### 4.1 Cell culture, primary hepatocyte isolation, and hepatocyte I/R modeling

The human hepatoma‐derived cell line HuH7, murine hepatocyte cell line AML12, and human embryonic kidney 293T cell line (HEK293T) were obtained from the Type Culture Collection of the Chinese Academy of Sciences (Beijing, China). PHCs were isolated from male mice aged 6–8 weeks, as previously described.^50^ To induce the IRI model in vitro, PHCs, HuH7, and AML12 cells were subjected to hypoxia (1% oxygen) in glucose‐free DMEM (Gibco) for 6 h, followed by culture under normoxic conditions for an additional 6 h.

### 4.2 Animals and treatments

Male mice (8–10 weeks old) with a C57BL/6 background were utilized for in vivo study. The mice were maintained in a specific pathogen‐free (SPF) facility under a controlled 12‐h light/dark cycle with access to chow and water ad libitum. A 75% hepatic warm IRI model was induced in the mice, as previously described.^51^ The in vivo animal experiments conducted in this study consisted of two parts: pharmacodynamic experiments and rescue experiments. Detailed information is presented in the Supporting Information.

### 4.3 qPCR, RNA‐seq, and data processing

Total RNA was extracted for cDNA synthesis using a HiScript III RT SuperMix for qPCR Kit (Vazyme) for qPCR analysis. Gene‐specific primers used were synthesized by Beijing Tsingke Biotech Co., Ltd., and their sequences are listed in Table S3. For the RNA‐seq assay, 250 ng of total RNA extracted from each liver sample and HuH7 cells were utilized to construct the cDNA library using an MGIEasy RNA Library Prep Kit (MGI Tech Co., Ltd.), followed by sequencing on a BGISEQ‐500 platform (MGI Tech Co.). See Supporting Information for detailed information on RNA‐seq.

### 4.4 Western blot and co‐immunoprecipitation assay

Liver samples and cultured cells were lysed to extract the total protein using RIPA lysis buffer supplemented with protease inhibitor cocktail (04693132001; Roche) and phosphatase inhibitors (4906845001; Roche). The extraction of membrane and cytosol proteins was conducted using the Membrane and Cytosol Protein Extraction Kit (P0033; Beyotime) in accordance with the manufacturer's instructions. The β‐actin served as an internal control. The specific primary antibodies used in the experiment are detailed in Table S4. For immunoprecipitation assays, HuH7 cells were co‐transfected with the designated plasmid for 24 h, followed by OGD incubation and Rk2 treatment.

### 4.5 Isothermal titration calorimetry assay

Calorimetric measurements of the interaction between Rk2 and human recombinant AKT1 were conducted using a Nano ITC calorimeter (TA Instruments). The standard NanoAnalyze software package (version 3.5.0) was utilized to process the data obtained from the ITC assay. Detailed information is presented in the Supporting Information.

### 4.6 Data analysis

Statistical analysis of all data in this study was conducted using SPSS statistical software (version 22.0, SPSS Inc.). For comparisons between two groups, the Student's *t*‐test or a nonparametric test was employed, while multiple‐group data comparisons were analyzed using one‐way analysis of variance followed by Bonferroni's multiple comparisons test. A *p*‐value of <0.05 was considered statistically significant.

## AUTHOR CONTRIBUTIONS

**Hong Shen**: Methodology; validation; investigation; data curation; writing—original draft preparation. **Jiajun Fu**: Methodology; investigation; data curation. **Jiayue Liu**: Methodology; investigation; data curation; resources. **Toujun Zou**: Methodology; validation. **Kun Wang**: Validation; data curation. **Xiao‐Jing Zhang**: Methodology; supervision; conceptualization. **Jian‐Bo Wan**: Conceptualization; supervision; writing—reviewing and editing; funding acquisition. All authors read and approved the final manuscript.

## CONFLICT OF INTEREST STATEMENT

The authors declare no conflicts of interest.

## ETHICS STATEMENT

The animal study protocols were approved by the Animal Care and Use Committee of Renmin Hospital of Wuhan University (Approval No.: WDRM20221009A, Wuhan, China).

## Supporting information



Supporting Information

## Data Availability

The raw RNA‐seq data of this study are available in the Gene Expression Omnibus (GEO) database (GSE284059 and GSE284060). All data generated or analyzed during this study are available upon reasonable request from the corresponding author.
