# Supplementary material for: Ginsenoside Rk2 alleviates hepatic ischemia/reperfusion injury by enhancing AKT membrane translocation and activation
Source: MedComm (2020). 2025 Jan 14;6(1):e70047. doi: 10.1002/mco2.70047 (PMC11731106; doi:10.1002/mco2.70047)
Supplement: Supplementary file 1 — Supporting Information [file MCO2-6-e70047-s001.docx]

**Supplementary** **Materials**

**Ginsenoside Rk2 alleviates** **hepatic** **ischemia reperfusion-injury by enhancing AKT membrane translocation and activation**

Hong Shen^1^, Jiajun Fu^2^, Jiayue Liu^1^, Toujun Zou^3^, Kun Wang^2^, Xiao-Jing Zhang^2,4,^*, Jian-Bo Wan^1,^*

**^1^** State Key Laboratory of Quality Research in Chinese Medicine, Institute of Chinese Medical Sciences, University of Macau, Macao SAR, China

**^2^** State Key Laboratory of New Targets Discovery and Drug Development for Major Diseases, Gannan Innovation and Translational Medicine Research Institute, Gannan Medical University, Ganzhou, China

**^3^** Department of Cardiology, Renmin Hospital of Wuhan University, Wuhan, China

**^4^** Basic Medical School, Wuhan University, Wuhan, China

***Correspondences:**

**Prof. Jian-Bo Wan,** E-mail: jbwan@um.edu.mo

Room 6034, Building N22, Institute of Chinese Medical Sciences,

University of Macau, Avenida da Universidade, Taipa, Macao SAR, China

**Prof. Xiao-jing Zhang**

E-mail: zhangxjing@whu.edu.cn;

Basic Medical School, Wuhan University, Wuhan, China

**Table of Contents**  Pages

1. Supporting Information Text S1-S6

2. Supporting Tables (Table S1- Table S6) S7-S10

3. Supporting Figures (Figure S1- Figure S8) S11-S17

**Supporting** **Information Text**

**1. Chemicals and reagents**

Ginsenosides (**Table S1**) with purities exceeding 98% were obtained from Baoji Herbest Bio-Tech Co., Ltd. (Baoji, China) and Chengdu Alfa Biotechnology Co., Ltd. (Chengdu, China) for the screening test. LY294002 (CAS No. 154447-36-6), an inhibitor of phosphoinositide 3-Kinase and serine/threonine kinase AKT, was purchased from Shanghai Yuanye Biotechnology Co., Ltd. (Shanghai, China). Cremophor Oil (C5135) was sourced from Sigma-Aldrich (St. Louis, MO, USA).

**2. Cell culture and treatments**

PHCs, HuH7, and HEK293T cells were cultured in DMEM supplemented with 10% fetal bovine serum (FBS, Gibco), while AML12 cells were cultured in DMEM/Ham’s F12 (Gibco) with 10% FBS, dexamethasone insulin, selenium, and transferrin. All cell cultures were maintained in a humidified atmosphere of 5% CO_2_ at 37 ^o^C. PHCs and HuH7 cells were treated with DMEM containing DMSO or ginsenosides for 6h after hypoxic incubations (during the reoxygenation period). LY294002, dissolved in DMSO, was administered to PHCs and HuH7 cells for 18 hours prior to OGD/R.

**3. Cell viability and cell apoptosis assay**

Cell viability was assessed using the Cell Counting Kit-8 (CCK-8) assay (Bimake, Houston, TX, USA). The cell apoptosis assay was conducted using an Annexin V-FITC/PI Apoptosis Detection Kit (Yeasen, Shanghai, China) for PHCs and Annexin V-PE/7-AAD Apoptosis Detection Kit (Vazyme, Nanjing, China) for HuH7 and AML12 cells, in accordance with the manufacturer's instructions. Stained PHCs, HuH7 and AML12 cells were detected and analyzed using a confocal laser scanning microscopy (Zeiss, Germany) and a CytoFLEX flow cytometer (Beckman Coulter, IN, USA), respectively.

**4. Luciferase assays**

The dual luciferase reporter assay system (Promega Corporation, Madison, WI, USA) was employed to measure the luciferase activity of both TNF and IL-6. Briefly, the pGMLR-TK plasmid was co-transfected with TNFα- and IL6-promoter luciferase reporter plasmids in HuH7 cells using Lipofectamine^TM^ 2000 reagent. Following treatments and I/R incubation, HuH7 cells were harvested and lysed to sequentially measure the luciferase activities of Firefly and Renilla.

**5. Mouse hepatic IRI modeling and treatments**

A 75% hepatic warm IRI model was induced in the mice for evalution . After anesthetization with sodium pentobarbital, a midline laparotomy was performed to expose the liver. The left and middle portal vein branches were clamped using an atraumatic microvascular clamp for 1 h to induce hepatic ischemia, followed by the 6 h of reperfusion for. Sham-operated mice underwent the same surgical procedure without vascular occlusion to serve as controls. Successful modeling of I/R was indicated by significant liver injury, evidenced by elevated serum levels of ALT and AST, along with the presence of hepatic necrosis.

The *in vivo* animal experiments conducted in this study consisted of two parts: pharmacodynamic experiments and rescue experiments. For pharmacodynamic experiments, mice were randomly assigned to five groups after a week of acclimatization, *i.e.*, sham group (sham, *n* = 10), sham plus Rk2 treatment group (sham+Rk2, 30 mg/kg, *n* = 10), I/R group (*n* = 12), I/R plus Rk2 treatment groups (I/R+LRk and I/R+HRk; 10 mg/kg and 30 mg/kg, *n* = 12 per group). For the rescue experiments, mice were randomly assigned into 6 groups, *i.e.*, sham group (*n* = 10), sham plus LY294002 group (sham+LY; 35 mg/kg, *n* = 10), I/R group (*n* = 12), I/R plus LY294002 treatment group (I/R+LY; 35 mg/kg, *n* = 12), I/R plus Rk2 treatment group (I/R+Rk; 30 mg/kg, *n* = 12)), I/R plus LY294002 and Rk2 group (I/R+LY+Rk; 35 mg/kg and 30 mg/kg respectively, *n* = 12). The mice in the I/R groups were underwent hepatic IRI, while those in other groups were subjected to sham operation. Mice from the sham and I/R alone group received intraperitoneal injections of a vehicle solution composed of 5% dimethyl sulfoxide, 10% castor oil, and saline. In contrast, the Rk2-treated and LY294002-treated groups received intraperitoneal injections of Rk2 and LY294002, respectively, both of which were dissolved in the vehicle solution. These injections were administered two days prior to the surgery and at the initiation of perfusion. Upon completion of the reperfusion period, the surviving mice were immediately euthanized using sodium pentobarbital to collect blood and liver tissues. During the pharmacodynamic experiments, one mouse, two mice, and one mouse died in the I/R, I/R+LRk, and I/R+HRk groups, respectively. In the rescue experiments, one mouse, one mouse, two mice, and one mouse died in the I/R, I/R+LY, I/R+Rk, and I/R+LY+Rk groups, respectively. Serum ALT and AST levels were assessed to evaluate the liver injury using an ADVIA 2400 Chemistry System Analyzer (Siemens, NY, USA) following the manufacturer's protocols. Partial liver tissue specimens were preserved in a 10% neutral formalin fix solution for histological assessments. The remaining liver tissue samples were rapidly frozen in liquid nitrogen for the examination of protein and gene expressions.

**6. Plasmid construction, cell transfection and stable cell line construction**

Human TNFα- and IL6-promoter luciferase reporter plasmids, as well as pGMLR-TK luciferase reporter plasmids were acquired from Yeasen Biotechnology Co., Ltd. (Shanghai, China). The full-length human AKT1 and PDPK1 genes were amplified from human cDNA and cloned into the PHAGE vector. Additionally, a human AKT1 mutant plasmid (where lysine 14 was mutated to arginine) was constructed by reversing the expansion of the full-length plasmid. Knockdown sequences (**Table S2**) for human AKT1 and mouse Akt1 were designed and incorporated into the PLKO.1-HIV-based lentiviral vectors. All plasmid DNA sequences were verified by sequencing performed by Tsingke (Beijing, China). Cells were transfected with the designated plasmids using a Lipofectamine^TM^ 2000 reagent and harvested 24 h after transfection for further analysis. Stable cell lines exhibiting overexpression of wild-type human AKT1 and the AKT (K14R) mutant, as well as knockdown of AKT1, RICTOR, and Akt1 expression in HuH7 and AML12 cells, were generated by infection with packaged lentiviruses that produced in HEK293T cells by transfection the target genes plasmids along with packaging plasmids psPAX2 and pMD2.G. Following infection, the cells were treated with puromycin to select for stable cell lines of HuH7 and AML12, respectively.

**7. Immunofluorescence and histology staining**

Following OGD/R incubation and treatment with Rk2, the PHCs and HuH7 cells were fixed, permeabilized, and blocked, followed by incubation overnight at 4 °C with primary antibodies, including anti-HA, anti-Akt, anti-ATP1A1, and anti-phospho-Akt (Ser473). Afterward, the cells were incubated in the dark for 1 h with the corresponding secondary antibody. Following DAPI staining, the expression and subcellular localization of phospho-Akt and exogenous HA-tagged AKT1 were observed using a confocal laser scanning microscopy (Zeiss).

For the visual assessment of pathological changes in the ischemia-reperfused area of the mouse livers, paraffin-embedded liver sections (5 µm thick) were subjected to dewaxing, rehydration, and staining with H&E. Additionally, H&E staining was performed on lungs, liver, spleen, and kidneys for histopathological safety evaluation of Rk2 treatments in mice. Immunohistochemistry staining was conducted to analyze CD11b^+^ macrophage infiltration in the ischemia-reperfused area of the mouse livers. Briefly, after antigen retrieval and blocking, liver sections were incubated overnight at 4 ^o^C with a rabbit anti-CD11b^+^ primary antibody (1:4000 dilution; Boster, Wuhan, China), followed by incubation with anti-rabbit immunoglobulin and diaminobenzidine staining according to the instructions provided in Rabbit Two-step Detection Kit (ZSGB-BIO, Beijing, China). To visualize hepatocyte apoptosis induced by IRI, terminal deoxynucleotidyl transferase deoxyuridine triphosphate nick end labeling (TUNEL) staining was performed following the manufacturer’s instructions (Servicebio, Wuhan, China). Images were quantitatively analyzed using Image J^®^ software.

**8. qPCR, RNA-seq and data processing**

qPCR was performed with ChamQ SYBR qPCR Master Mix (Q311-03, Vazyme) on a LightCycler 480 Instrument (Roche, Switzerland). The mRNA expression values were normalized to β-actin. For the RNA-seq assay, 250 ng of total RNA extracted from each liver sample and HuH7 cells were utilized to perform RNA-seq. To analyze the raw sequencing data, HISAT2 (version 2.21), SAMtools (version 1.4), and StringTie (version 1.3.3b) were employed. The differential gene expression was analyzed using the DESeq2 package, while the KEGG pathway and GSEA enrichment analysis were conducted using the R package “clusterProfiler” to assess the variation in pathway activity under different conditions. Gene sets with *P* values of < 0.05 and false discovery rate (FDR) values of < 0.25 were defined as statistical significance.

**9. Western blot and co-immunoprecipitation assay**

Western blot assays were conducted on both liver samples and cultured cells. Following the processes of lysation and denaturation, the protein-SDS mixture was separated electrophoretically on 10-15% SDS-PAGE gels and subsequently transferred to PVDF membranes (Millipore). These membranes were blocked with 5 % BSA for 60 min at room temperature and then incubated overnight at 4 ^o^C with appropriate primary antibodies, followed by incubation with the corresponding secondary antibodies for 1 h at room temperature. Images were captured and analyzed using a ChemiDoc MP Imaging System (Bio-Rad, Hercules, CA, USA).

For immunoprecipitation (IP) assays, HuH7 cells were lysed with an ice-cold IP buffer supplemented with a protease inhibitor cocktail after OGD incubation and Rk2 treatment. After preparing the whole cell lysate as the input control, the remaining lysate was incubated with protein A/G agarose beads (Roche) along with the corresponding antibody (anti-Flag or anti-HA) in rotation for 6 h at 4 ^o^C. The beads were then thoroughly washed and boiled in 2× SDS loading buffer at 95 °C for 10 min. Western blot analysis was conducted as previously above.

**10.** **Network pharmacology analysis**

For the network pharmacology analysis of the pharmacological effects of ginsenosides, all target genes associated with their pharmacological activities, both *in vitro* and *in vivo*, were retrieved from the Comparative Toxicogenomics Database. Enrichment analysis, including disease annotation of the Disease Gene Network, Gene Ontology, and KEGG pathways, were performed using the tools provided by Metascape (https://metascape.org).

To identify Rk2 targets in the treatment of hepatic IRI, target information for Rk2 was obtained from the PharmMapper and chEMBL databases based on the chemical structure data. Meanwhile, disease targets related to hepatic IRI were collected from the DisGeNET and GeneCards databases. Venn analysis was performed to identify overlap between the predicted gene/protein targets of Rk2 and disease targets associated to hepatic IRI using an online tool (<http://bioinformatics.psb.ugent.be/> webtools/Venn/) for visualization. Subsequently, a protein-protein interaction (PPI) network was constructed using the STRING database. The resulting data were visualized and analyzed by using Cytoscape 3.10 and its analysis plugin, cytoHubba. Topological analysis based on maximum clique centrality values was employed to identify core genes.

**11. Literature reviews of studies on the treatment of IRIs with ginsenosides** **and their derivatives**

MEDLINE (PubMed) and Web of Science databases were searched to identify articles published between January 2000 and January 2024 using the MeSH terms and the free text terms including "reperfusion injury," "ginsenoside," "notoginsenoside," "gypenoside," "ginseng," " Panax ginseng," "Panax notoginseng," and "notoginseng." Duplicate studies, review articles, case reports, conference abstracts or posters were excluded, with 42 studies being included for data analysis. Following the extraction of data regarding the pharmacological treatment of IRIs, a preliminary analysis of the current status of ginsenosides and their derivatives in the treatment of IRIs was conducted using bibliometric methods.

**12. Molecular docking**

Molecular docking studies were carried out using the Glide module of Schrodinger software (Schrödinger Release, 2021). The two-dimensional (2D) structure of ginsenoside Rk2, obtained from the PubChem database, was imported into the software and converted into a three-dimensional (3D) structure for docking analysis. Crystal structures of human AKT1 (PDB code: 6S9W), CASP3 (PDB code: 5IBP), EGFR (PDB code: 8A27), ALB (PDB code: 6YG9), and MMP9 (PDB code: 4XCT) were retrieved from the RCSB Protein Data Bank (RCSB PDB) and prepared for docking by adding hydrogen atoms and assigning charges using the Protein Preparation Wizard module. To facilitate the docking process, optimal binding sites of the proteins were predicted and enclosed within appropriate boxes using Schrodinger's SiteMap and Receptor Grid Generation module. These boxes defined the active pockets for molecular docking between the ligand and receptor. The docking procedure was then performed using the Glide module. Binding affinity was evaluated by calculating the molecular mechanics generalized Born surface area (MM/GBSA) and the XP Gscore. To visualize the binding sites and interactions between the ligand and receptor, images were generated using PyMOL, illustrating the nature of these interactions.

**13.** **Isothermal Titration Calorimetry (ITC) assay**

Human recombinant AKT1 was prepared as a His-tagged fusion protein. The coding sequence of AKT1 was cloned into a pET28a vector and transformed into *Escherichia coli* BL21 star (DE3) cells, followed by induction with isopropyl β-d-thiogalactopyranoside (IPTG). The His-tagged AKT1 fusion protein was then purified using a single-step affinity chromatography. Calorimetric measurements of the interaction between Rk2 and human recombinant AKT1 were conducted at 25°C using a Nano ITC calorimeter (TA Instruments, New Castle, DE, USA). Solutions of Rk2 and human recombinant AKT1 protein were prepared in PBS buffer containing 10 % DMSO at the specified concentration. A water-to-water titration was performed prior to each experiment to ensure measurement accuracy. Initially, the sample cell was filled with a vehicle solution and titrated with 1 mM Rk2 solution to establish a background titration. Subsequently, 50 μL of the Rk2 solution from the syringe was incrementally injected into the sample cell, which contained a dissolved protein solution at a concentration of 0.024 mM. The stirring rate of the paddle was set at 350 rpm, and the time interval between injections was empirically set at 120 s, allowing sufficient time for the signal to return to the baseline. The standard NanoAnalyze software package (version 3.5.0) was utilized to process the data obtained from the ITC assay.

**Supporting Tables**

**Table S1.**Ginsenosides for screening of potentially effective agents for Hepatic IRI

| **No.** | **Compounds** | **CAS NO.** | **M.F.** | **Natural abundance** |
| --- | --- | --- | --- | --- |
| **1** | 20(S)-Protopanaxadiol (PPD) | 30636-90-9 | C_30_H_52_O_3_ | Rare |
| **2** | [(20S)-Protopanaxatriol](https://pubchem.ncbi.nlm.nih.gov/compound/11468733) (PPT) | [34080-08-5](https://pubchem.ncbi.nlm.nih.gov/compound/11468733) | C_30_H_52_O_4_ | Rare |
| **3** | Ginsenoside F1 | 53963-43-2 | C_36_H_62_O_9_ | Rare |
| **4** | Ginsenoside Ra1 | 83459-41-0 | C_58_H_98_O_26_ | Rare |
| **5** | Ginsenoside Rc | 11021-14-0 | [C_53_H_90_O_22_](https://pubchem.ncbi.nlm.nih.gov/#query=C53H90O22) | Macro |
| **6** | Ginsenoside Re | 51542-56-4 | C_48_H_82_O_18_ | Macro |
| **7** | Ginsenoside Rf | 52286-58-5 | C_42_H_72_O_14_ | Rare |
| **8** | Ginsenoside Rg1 | [22427-39-0](https://pubchem.ncbi.nlm.nih.gov/compound/441923) | [C_42_H_72_O_14_](https://pubchem.ncbi.nlm.nih.gov/#query=C42H72O14) | Macro |
| **9** | Ginsenoside Rg3 | 14197-60-5 | C_42_H_72_O_13_ | Rare |
| **10** | Ginsenoside Rg5 | 186763-78-0 | C_42_H_70_O_12_ | Rare |
| **11** | Ginsenoside Rh2 | [78214-33-2](https://pubchem.ncbi.nlm.nih.gov/compound/119307) | C_36_H_62_O_8_ | Rare |
| **12** | Ginsenoside Rh3 | 105558-26-7 | C_36_H_60_O_7_ | Rare |
| **13** | Ginsenoside Rk1 | 494753-69-4 | C_42_H_70_O_12_ | Rare |
| **14** | Ginsenoside Rk2 | 364779-14-6 | C_36_H_60_O_7_ | Rare |
| **15** | Notoginsenoside Fc | 88122-52-5 | C_58_H_98_O_26_ | Rare |
| **16** | Notoginsenoside Fd | 80321-63-7 | C_47_H_80_O_17_ | Rare |
| **17** | Notoginsenoside Fe | 88105-29-7 | C_47_H_80_O_17_ | Rare |
| **18** | Notoginsenoside Fp2 | 1004988-75-3 | C_58_H_98_O_26_ | Rare |
| **19** | Notoginsenoside R1 | 80418-24-2 | C_47_H_80_O_18_ | Rare |
| **20** | Notoginsenoside R4 | 87741-77-3 | C_59_H_100_O_27_ | Rare |
| **21** | Notoginsenoside S | 575446-95-6 | [C_63_H_106_O_30_](https://pubchem.ncbi.nlm.nih.gov/#query=C63H106O30) | Rare |
| **22** | Gypenoside Ⅸ | 80321-63-7 | C_47_H_80_O_17_ | Rare |
| **23** | Gypenoside XIII | 80325-22-0 | C_41_H_70_O_12_ | Rare |
| **24** | Gypenoside XVII | 80321-69-3 | C_48_H_82_O_18_ | Rare |

**Table S2.** shRNA sequences for the targeted genes used in this study

| **Knockdown constructs** | **Species** | **Target sequences (5'→ 3')** |
| --- | --- | --- |
| sh*AKT1* | Human | GGACTACCTGCACTCGGAGAA |
| sh*Akt1* | Mouse | TCTGAGACTGACACCAGGTAT |
| sh*RICTOR* | Human | CGGAGGTTCATACAAGAATTA |
| shCtrl | - | CAACAAGATGAAGAGCACCAA |

**Table S3.** Sequences of primers for quantitative real-time PCR

| **Genes** | **Species** | **Forward primer** (5'→ 3') | **Reverse primer**(5'→ 3') |
| --- | --- | --- | --- |
| *β-ACTIN* | Human | CATGTACGTTGCTATCCAGGC | CTCCTTAATGTCACGCACGAT |
| *TNFA* | Human | GAGGCCAAGCCCTGGTATG | CGGGCCGATTGATCTCAGC |
| *IL1B* | Human | AGCTACGAATCTCCGACCAC | CGTTATCCCATGTGTCGAAGAA |
| *IL6* | Human | ACTCACCTCTTCAGAACGAATTG | CCATCTTTGGAAGGTTCAGGTTG |
| *CCL2* | Human | ATAGCAGCCACCTTCATTCCC | CAGCTTCTTTGGGACACTTGC |
| *CXCL10* | Human | GTGGCATTCAAGGAGTACCTC | TGATGGCCTTCGATTCTGGATT |
| *RICTOR* | Human | GCTAGGTGCATTGACATACAACA | AGTGCTAGTTCACAGATAATGGC |
| *β-Actin* | Mouse | GTGACGTTGACATCCGTAAAGA | GCCGGACTCATCGTACTCC |
| *Tnfα* | Mouse | CATCTTCTCAAAATTCGAGTGACAA | TGGGAGTAGACAAGGTACAACCC |
| *Il1b* | Mouse | CCGTGGACCTTCCAGGATGA | GGGAACGTCACACACCAGCA |
| *IL6* | Mouse | TAGTCCTTCCTACCCCAATTTCC | TTGGTCCTTAGCCACTCCTTC |
| *Ccl2* | Mouse | TACAAGAGGATCACCAGCAGC | ACCTTAGGGCAGATGCAGTT |
| *Cxcl10* | Mouse | ATGACGGGCCAGTGAGAATG | ATGATCTCAACACGTGGGCA |

**Table S4.** Primary and secondary antibodies used in immunoblot and immunofluorescence analysis

| **Primary antibody** | **MW(kDa)** | **Cat. NO.** | **Source** | **Dilution** | **Company** |
| --- | --- | --- | --- | --- | --- |
| Phospho-PI3K p85 (Tyr458)/p55(Tyr199) | 85 | 4228 | Rabbit | 1:1000 | Cell Signaling Technology |
| PI3K p85 | 60,85 | 4292 | Rabbit | 1:1000 | Cell Signaling Technology |
| phospho-PDPK1(Ser241) | 68 | sc-32960 | Rabbit | 1:200 | Santa Cruz Biotechnology |
| PDK1 | 68 | sc-17766 | Rabbit | 1:200 | Santa Cruz Biotechnology |
| PTEN | 54 | 9559 | Rabbit | 1:1000 | Cell Signaling Technology |
| phospho-AKT (Thr308) | 60 | 13038 | Rabbit | 1:1000 | Cell Signaling Technology |
| phospho-AKT(Ser473) | 60 | 4060 | Rabbit | 1:1000 | Cell Signaling Technology |
| AKT (pan) | 60 | 4691 | Rabbit | 1:1000 | Cell Signaling Technology |
| phospho-AKT(Ser473) monoclonal antibody | 60 | 66444-1-Ig | mouse | IF:1:300 | Proteintech |
| AKT (pan) monoclonal antibody | 60 | 60203-2-Ig | mouse | IF:1:400 | Proteintech |
| ATP1A1 | 100 | 14418-1-AP | Rabbit | IF:1:400; WB:1:2000 | Proteintech |
| phospho-FOXO1(Ser241) | 82 | 9461 | Rabbit | 1:1000 | Cell Signaling Technology |
| Phospho-GSK3β (Ser9) | 46 | 9323 | Rabbit | 1:1000 | Cell Signaling Technology |
| phospho-BAD (Ser136) | 23 | 4366 | Rabbit | 1:1000 | Cell Signaling Technology |
| BAD | 23 | 9292 | Rabbit | 1:1000 | Cell Signaling Technology |
| BAX | 20 | 2772 | Rabbit | 1:1000 | Cell Signaling Technology |
| BCL-2 | 26 | 4223 | Rabbit | 1:1000 | Cell Signaling Technology |
| cleaved CASP3 (Asp175) | 17,19 | 9664 | Rabbit | 1:1000 | Cell Signaling Technology |
| β-Actin | 42 | 81115-1-RR | Rabbit | 1:5000 | Proteintech |
| HA tag | 1 | 66006-2-Ig | Mouse | IF: 1:400; WB:1:5000 | Proteintech |
| HA tag | 1 | 51064-2-AP | Rabbit | 1:5000 | Proteintech |
| Flag tag | 1 | 66008-4-Ig | Mouse | 1:5000 | Proteintech |
| Flag tag | 1 | 20543-1-AP | Rabbit | 1:5000 | Proteintech |

**Table S5.** Top 5 core proteins in network string interactions ranked by MCC method.

| **Rank** | **Node Name** | **MCC** | **Degree** | **MNC** | **EPC** | **BottleNeck** | **EcCentricity** | **Closeness** | **Radiality** | **Betweenness** | **Stress** | **Clustering coefficient** |
| --- | --- | --- | --- | --- | --- | --- | --- | --- | --- | --- | --- | --- |
| 1 | AKT1 | 2.3518E+11 | 45 | 45 | 24.53 | 40 | 0.5 | 52 | 2.83 | 311.37 | 1808 | 0.42 |
| 2 | CASP3 | 2.3518E+11 | 40 | 40 | 24.37 | 1 | 0.5 | 49.5 | 2.75 | 178.5 | 1216 | 0.49 |
| 3 | EGFR | 2.3498E+11 | 35 | 35 | 24.07 | 3 | 0.3 | 46.7 | 2.63 | 124.26 | 826 | 0.54 |
| 4 | ALB | 2.3465E+11 | 45 | 45 | 24.73 | 6 | 0.5 | 52 | 2.83 | 344.06 | 1884 | 0.4 |
| 5 | MMP9 | 2.3465E+11 | 34 | 34 | 23.03 | 1 | 0.5 | 46.5 | 2.64 | 92.47 | 818 | 0.58 |

**Table S6.** Results of molecular docking of Rk2 with predicted targets in this study.

| **Compound** | **Target** | **PDB ID** | **XP Gscore** | **MM-GBSA dG Bind(kcal/mol)** |
| --- | --- | --- | --- | --- |
| Rk2 | AKT1 | 6S9W | -9.775 | -26.35 |
|  | CASP3 | 5IBP | -2.831 | -24.88 |
|  | EGFR | 8A27 | -3.586 | -40.92 |
|  | ALB | 6YG9 | -2.433 | -20.56 |
|  | MMP9 | 4XCT | -4.613 | -2.17 |

**Supplementary Figures**


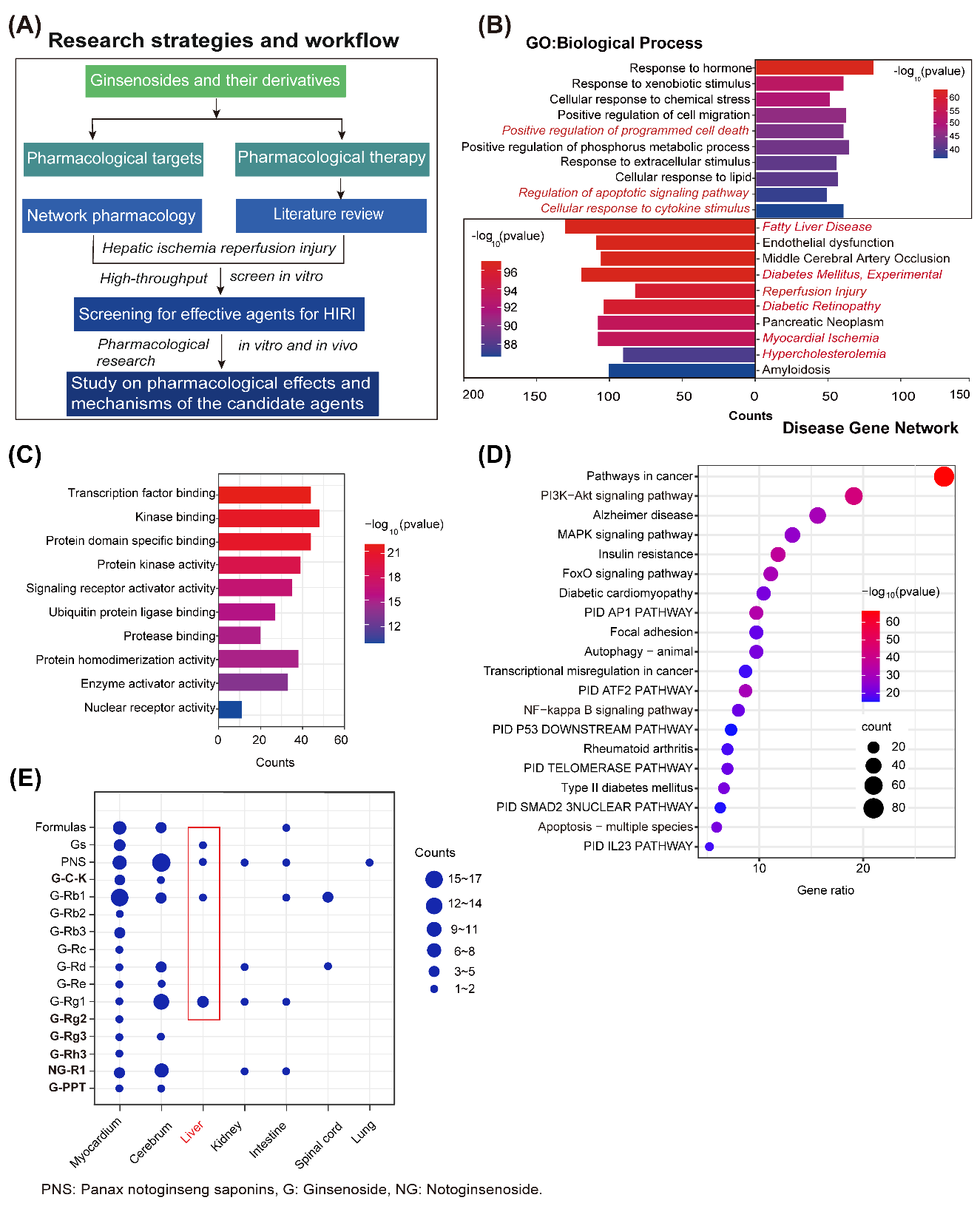


**Figure S1.** Network pharmacology analysis of the pharmacological mechanisms of ginsenosides and the summary of literature reviews on the pharmacological effects of ginsenosides in the treatment of IRIs. (A) Research strategy and workflow. (B) The top 10 terms of enrichment analysis of disease annotation of Disease Gene Network and GO annotation of biological processes on target genes of ginsenosides. (C) The top 10 terms of GO annotation enrichment analysis of molecular functions on target genes of ginsenosides. (D**)** The top 20 terms of KEGG pathway enrichment analysis on target genes of ginsenosides. (E) A summary of literature reviews on the pharmacological effects of ginsenosides and their derivatives in the treatment of IRIs.

**
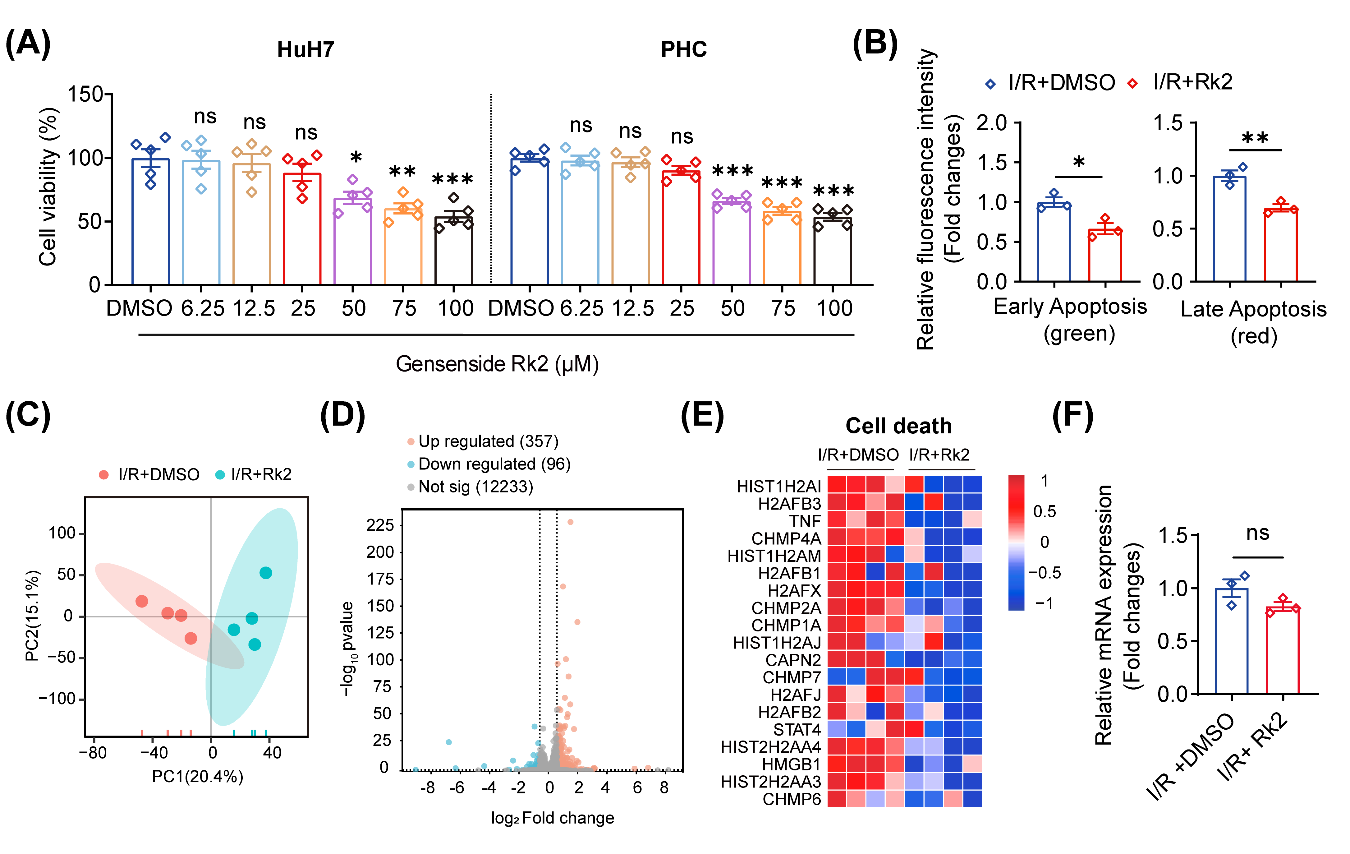
**

**Figure S2.** (A) Cell viability of HuH7 cells and PHCs under normal culture conditions after treatment with various concentrations of Rk2 for 24 h. Each value is shown as the mean ± SEM of five replicates. (B) The quantitative results of early and late apoptosis determined by analysing the fluorescence intensity in the green and red channels. Each value is shown as the mean ± SEM of three replicates. (C) Principal component analysis (PCA) of normalized RNA-seq expression values from HuH7 cells subjected to OGD/R incubation and treatment with or without Rk2 (20 μM). (D) A volcano plot illustrating differential expression of genes with statistical significance and fold change in HuH7 cells subjected to OGD/R incubation and treatment with or without Rk2 (20 μM). Significant genes were selected by fold change (>1.5- or < –1.5-fold) and adjusted *p*-value (<0.05). (E) The expression profile of genes related to the cell death in HuH7 cells subjected to OGD/R incubation and treatment with or without Rk2 (20 μM). (F) The mRNA levels of *CXCL10* in HuH7 cell lines after OGD/R incubation and treatment of Rk2 (20 μM). Each value is shown as the mean ± SEM of three replicates. **P* < 0.05, ***P* < 0.01, ****P* < 0.001, n.s., not significant, *vs.* DMSO-treated groups.

**
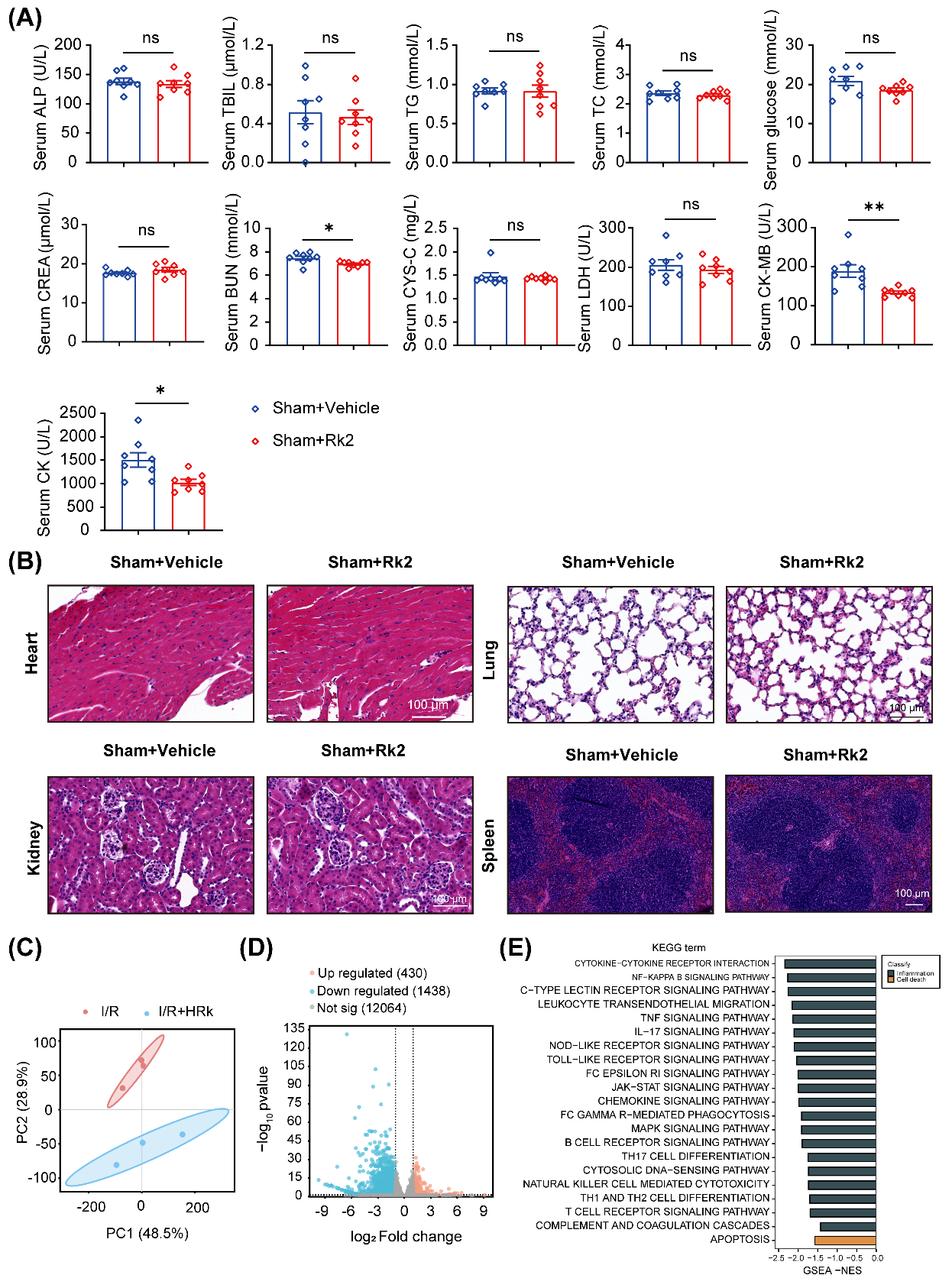
**

**Figure S3.** (A) Evaluation of biochemical parameters in mice (n = 8) from sham and sham + Rk2 groups (30 mg/kg). (B) Representative images of H&E staining of the heart, lung, spleen and kidney from the sham and sham + Rk2 groups (30 mg/kg). Values (n = 6) are shown as mean ± SEM; **P* < 0.05, ***P* < 0.01, n.s., not significant. **(**C**)** PCA of normalized RNA-seq expression values of liver samples from the I/R group and I/R+HRk group. (D) A volcano plot illustrating differential expression of genes with statistical significance and fold change in the I/R group and I/R+HRk group. Significant genes were selected by fold change (>2- or < –2-fold) and adjusted *p*-value (<0.05). (E) GSEA enrichment of differentially regulated pathways involved in inflammation and cell death in liver samples after hepatic I/R and Rk2 pretreatment in mice. TBIL, Total Bilirubin; ALP, alkaline phosphatase; TC, total cholesterol; TG, triglyceride; CREA, Creatinine; BUN, Blood Urea Nitrogen; CYS-C, Cystatin C; LDH, lactate dehydrogenase; CK, Creatine Kinase; CK-MB, Creatine kinase isoenzyme MB; GLU, Glucose.


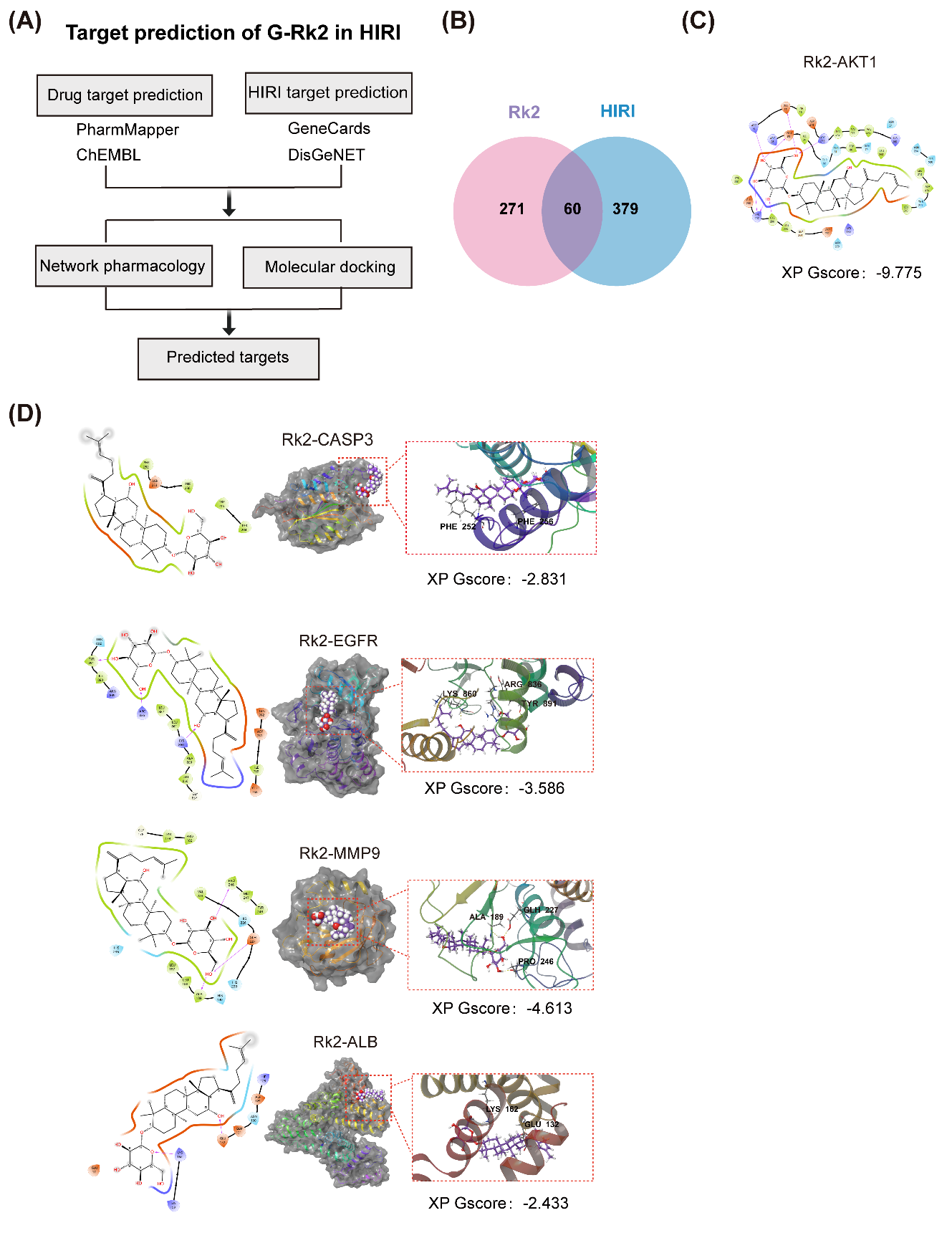


**Figure S4.** (A) Schematic representation of research protocol for the target identification of Rk2 in treating hepatic IRI. (B) Venn diagram depicting the overlap of targets for Rk2 and HIRI. (C and D) 2D and 3D molecular docking images showing the interaction between Rk2 and predicted targets AKT1, CASP3, EGFR, ALB and MMP9.


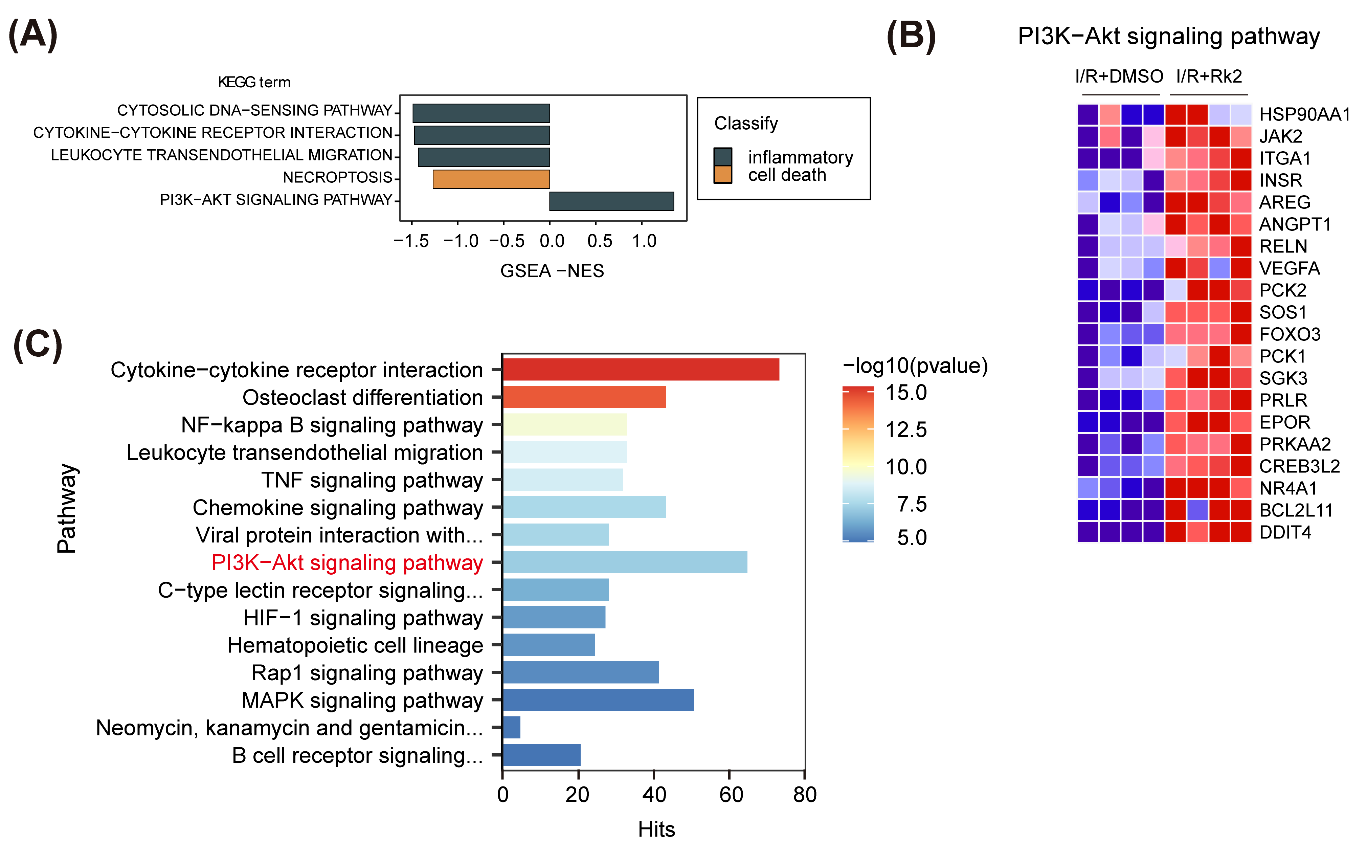


**Figure S5.** (A) GSEA enrichment of differentially regulated pathways involved in inflammation and cell death in HuH7 cells subjected to OGD/R incubation and treatment with Rk2 (20 μM). (B) A heat map of top 20 key genes in the activated PI3K-AKT signaling pathway based on GSEA enrichment in HuH7 cells subjected to OGD/R incubation and treatment with or without Rk2 (20 μM). (C) KEGG pathways enrichment analysis on differential expression genes between the I/R group and I/R+HRk group in mice.


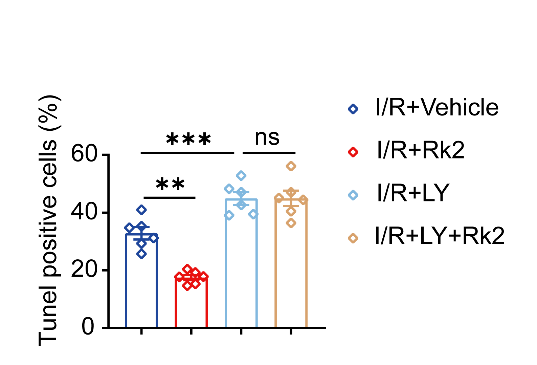


**Figure S6.** Statistical charts of TUNEL in liver sections of mice (n = 6).


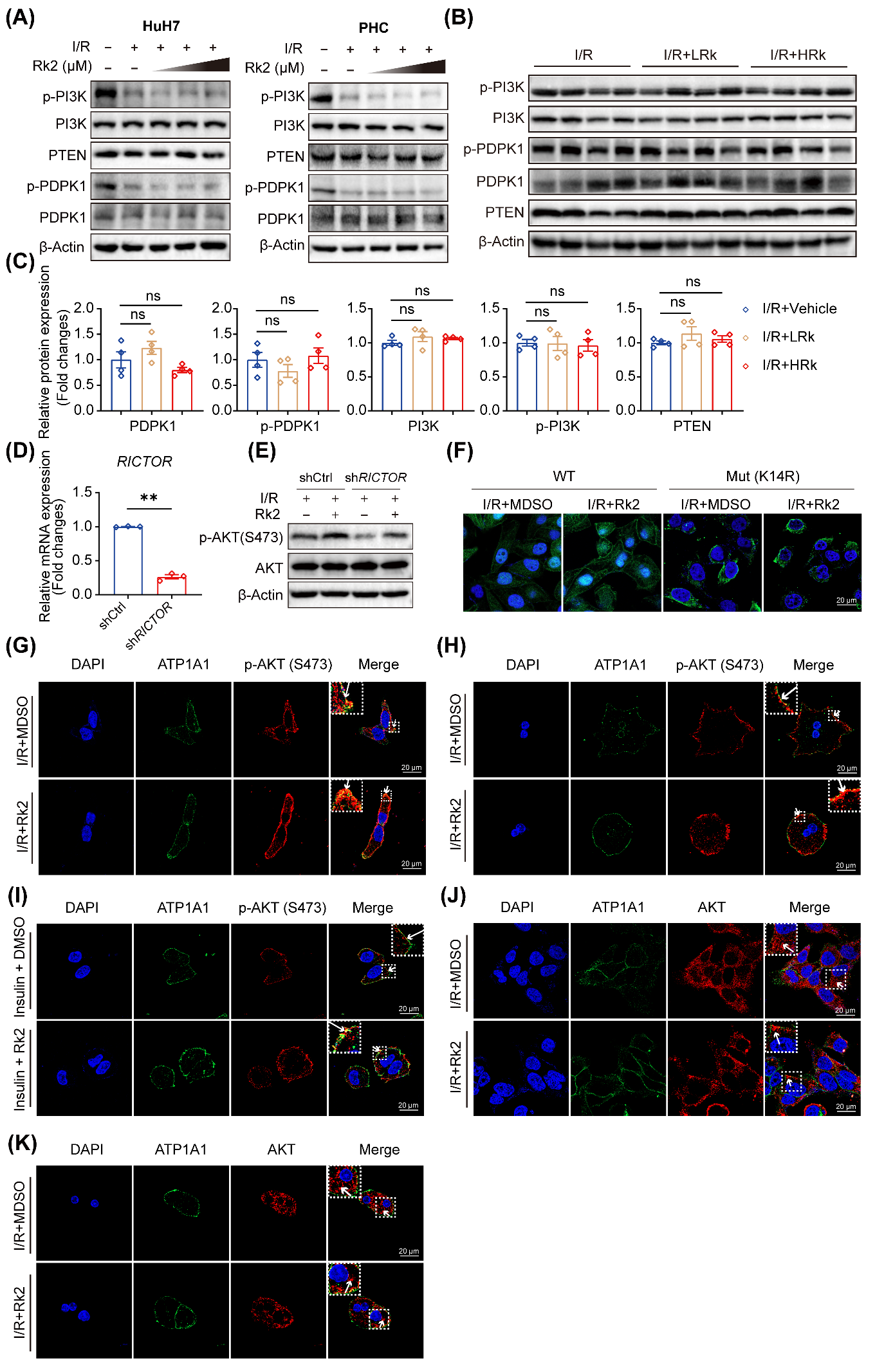


**Figure S7.** (A) Protein expression of p-PI3K, p-PDPK1, PI3K, PDPK, and PTEN in HuH7 cells (left) / PHCs (right) after OGD/R incubation and treatment with various concentrations of Rk2 (5 μM, 10 μM, 20 μM). (B and C) Western blot analysis (B) and the quantification (C, n = 4) of protein levels of PI3K, PDPK1, p-PI3K, p-PDPK1 and PTEN in liver tissue of mice. (D) The mRNA levels of *RICTOR* in HuH7 cell lines with endogenous *RICTOR* knockdown. Each value is shown as the mean ± SEM of three replicates. (E) Protein expression of p-AKT (S473) and total AKT in HuH7 cell lines with endogenous *RICTOR* after OGD/R incubation and treatment with Rk2 (20 μM). β-Actin served as the internal control. Data represent three separate experiments, and values are shown as mean ± SEM. (F) Representative fluorescence microscopy images of expression and subcellular localization of exogenous wild cherry-HA-tagged AKT1 and cherry-HA-tagged AKT (K14R) mutant in HuH7 cells. (G and H) Representative fluorescence microscopy images of expression and subcellular localization of endogenous p-AKT (Ser473) in HuH7 cells (left) / PHCs (right) after OGD/R incubation and treatment with Rk2 (20 μM). (I) Representative fluorescence microscopy images of expression and subcellular localization of endogenous p-AKT (Ser473) in HuH7 cells after treatment with Rk2 (20 μM) followed by insulin stimulation (100 nM, 10 min). (J and K) Representative fluorescence microscopy images of expression and subcellular localization of endogenous AKT in HuH7 cells (up) and PHCs (down) after OGD/R incubation and treatment with Rk2 (20 μM). n.s., not significant. **P* < 0.05, ***P* < 0.01, ****P* < 0.001, n.s., not significant.


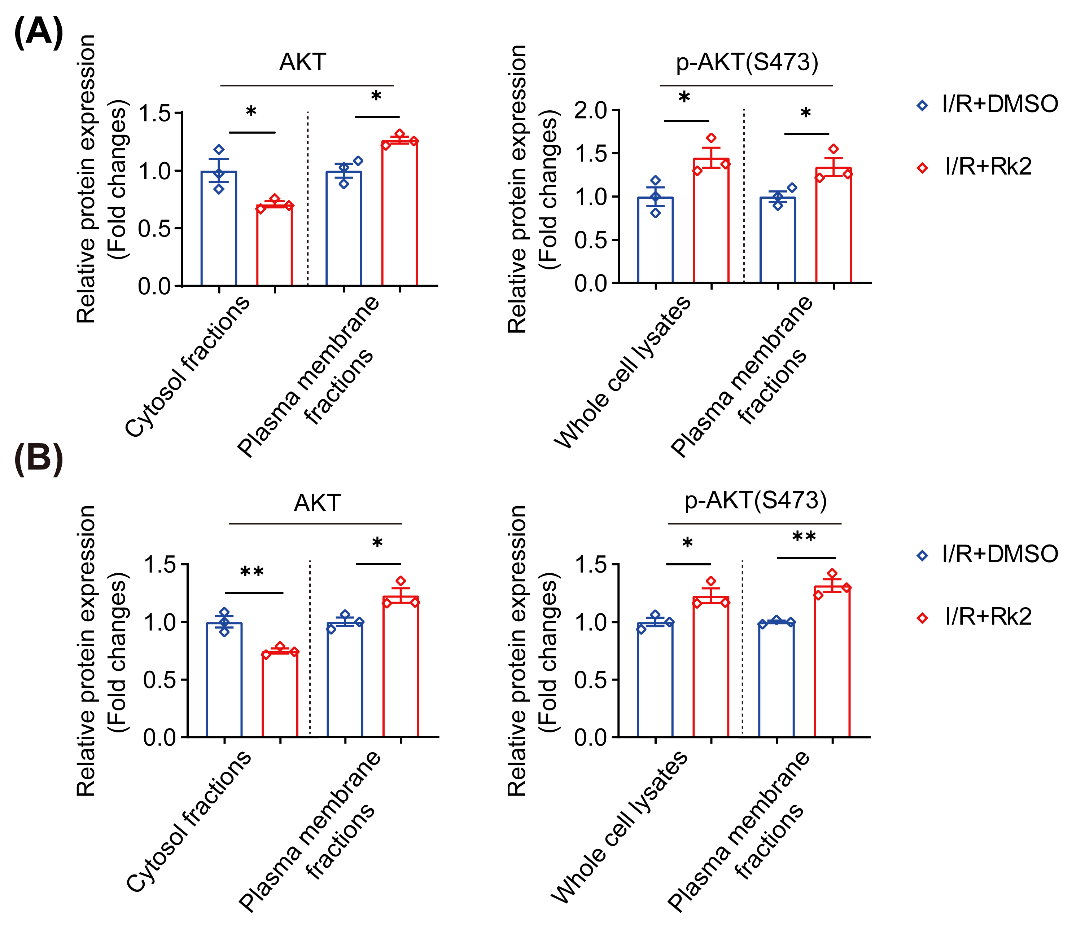


**Figure S8.** (A and B) Western blot quantification of protein expression of total AKT and p-AKT (Ser473) in whole cell lysates, cytosol fractions and plasma membrane fractions in HuH7 cells (A) / PHCs (B) after OGD/R incubation and treatment with Rk2 (20 μM). β-Actin and ATP1A1 were used as internal controls for cytoplasmic proteins and membrane proteins, respectively. **P* < 0.05, ***P* < 0.01, ****P* < 0.001. Each value is shown as the mean ± SEM of three replicates.
